# The “Bubblepole” (BUPO) Method for Linear-Scaling Coulomb Matrix Construction with or without Density Fitting

**DOI:** 10.1021/acs.jpca.4c07415

**Published:** 2025-03-03

**Authors:** Frank Neese, Pauline Colinet, Bernardo DeSouza, Benjamin Helmich-Paris, Frank Wennmohs, Ute Becker

**Affiliations:** † Department of Molecular Theory and Spectroscopy, Max-Planck-Institut für Kohlenforschung, D-45470 Mülheim an der Ruhr, Germany; ‡ FAccTs GmbH, Rolandstraße 67, 50667 Köln, Germany

## Abstract

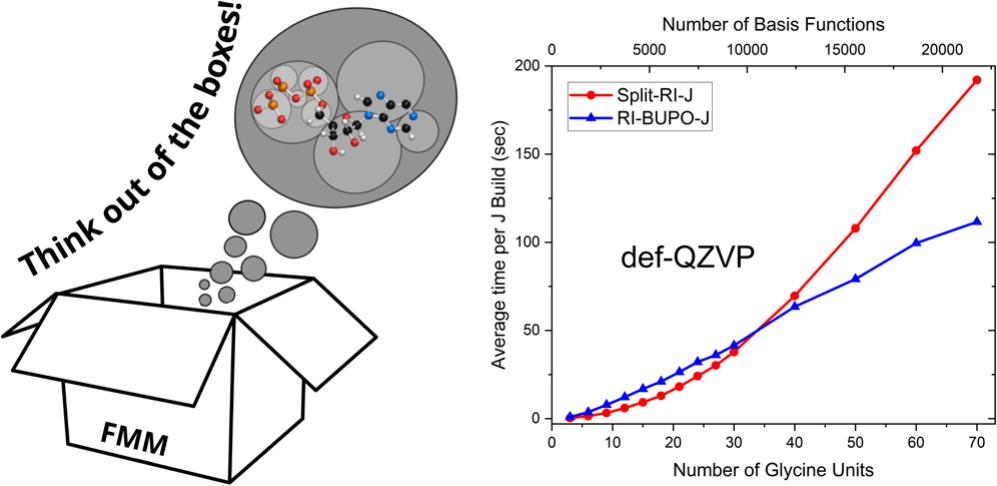

In this work, we describe the development of a new algorithm for the computation of Coulomb-type matrices using the well-known resolution of the identity (RI) or density fitting (DF) approximation. The method is linear-scaling with respect to system size and computationally highly efficient. For small molecules, it performs almost as well as the Split-RI-J algorithm (which might be the most efficient RI-J implementation to date), while outperforming it for larger systems with about 300 or more atoms. The method achieves linear scaling through multipole approximations and a hierarchical treatment of multipoles. However, unlike in the fast multipole method (FMM), the algorithm does not use a hierarchical boxing algorithm. Rather, close-lying objects like auxiliary basis shells and basis set shell pairs are grouped together in spheres that enclose the set of objects completely, which includes a new definition of the shell-pair extent that defines a real-space radius outside of which a given shell pair can be safely assumed to be negligible. We refer to these spheres as “bubbles” and therefore refer to the algorithm as the “Bubblepole” (BUPO) algorithm, with the acronym being RI-BUPO-J. The bubbles are constructed in a way to contain a nearly constant number of objects such that a very even workload arises. The hierarchical bubble structure adapts itself to the molecular topology and geometry. For any target object (shell pair or auxiliary shell), one might envision that the bubbles “carve” out what might be referred to as a “far-field surface”. Using the default settings determined in this work, we demonstrate that the algorithm reaches submicro-Eh and even nano-Eh accuracy in the total Coulomb energy for systems as large as 700 atoms and 7000 basis functions. The largest calculations performed (the crambin protein solvated by 500 explicit water molecules in a triple-ζ basis) featured more than 2000 atoms and more than 33,000 basis functions.

## 1 Introduction

It is probably fair to state that the majority of CPU hours consumed by electronic-structure calculations to date are used to perform self-consistent field (SCF) iterations, mostly based on some flavor of density functional theory (DFT). Given that these calculations are time-consuming (compared to, say, semiempirical calculations or force-field calculations), it is important to look for the most efficient implementations possible. Indeed, impressive progress has been made during the last two to three decades. While back then calculations on systems with hundred atoms were challenging, to date calculations with thousand atoms are definitely feasible. Even calculations with as many as ten-thousand atoms, previously firmly in the domain of force-field calculations, appear to be within reach.

If one reaches the domain of thousands of atoms, the spatial extent of the investigated systems is necessarily large and reaches dozens, if not hundreds of Angström diameter. This allows for techniques that scale linearly with system size to become effective.^1,2^ The field of linear-scaling, or O(*N*)-scaling (N being a measure of system size), SCF calculations has been highly active since the 1990s.

Looking at the components of the total DFT energy, it quickly became clear that the numerical integration required to efficiently compute the exchange-correlation (XC) potential is readily organized in a linear-scaling fashion.^3^ The one-electron integrals necessary for building the Fock- or Kohn–Sham (KS) matrix do not change during the SCF calculations and have such a small prefactor that their calculation using traditional, nonlinear-scaling algorithms does not present a computational bottleneck, even for very large systems. However, linear-scaling algorithms for the computations of the overlap- and related matrices are well-known too.^4^

For the exact exchange, linear-scaling algorithms have been devised early on and their efficiency has been demonstrated.^5,6^ However, these terms still have a large prefactor and additional savings can be realized by alternative techniques such as the pseudospectral (PS)^7−10^ or the closely related chain-of-spheres (COSX) method,^11−13^ for which linear-scaling and sub-mEh accuracy has also been demonstrated.

The most difficult term to linearize is therefore the Coulomb interaction. It closely resembles the classic electrostatic interaction between two charge distributions, which may be written as
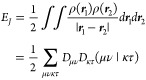
1where ρ(***r***) = ∑_μν_*D*_μν_μ(***r***)ν(***r***) is the electronic charge density, ***D*** is the density matrix, *E*_*J*_ is the Coulomb interaction energy and (μν|κτ) is an electron–electron repulsion integral in Mulliken notation. Clearly, the natural scaling of the Coulomb problem is quadratic and this is readily achieved in standard direct SCF calculations using prescreening techniques.^14−17^

The linearization of the Coulomb interaction has long been known to be possible on the basis of fast-multipole methods (FMMs). These techniques were first explored in quantum chemistry in pioneering work by Head-Gordon and co-workers^18,19^ and later been followed up by a number of authors.^20−26^ These techniques use a hierarchical boxing algorithm to divide real-space into boxes of equal size. The multipole expansion of the charge density inside each box allows for the presummation of the contributions of all charge sources in a given box. These can subsequently be used together with a “well-separatedness” criterion^18,19,27^ in order to obtain accurate approximations to the elements of the Coulomb matrix. If this is done carefully, the calculation of the Coulomb matrix can be organized into a “near-field” (NF) and a “far-field” (FF) contribution. The NF contains all interacting charge distributions that do not satisfy the well-separatedness criterion while the FF contains the remaining interactions. The number of significant shell pairs in an electronic-structure calculation scales linearly with system size, which is readily determined from the Gaussian product theorem (GPT).^28^ Consequently, the NF contributions also naturally scale linearly with system size given that there is an asymptotically constant number of next and perhaps second-next neighbors to a given shell pair that overlap with the shell pair itself. The calculation of the FF contribution would scale quadratically with system size, but can be made to scale linearly by using a recursive, hierarchical boxing algorithm.^18,19,28^

FMM-based algorithms for the calculation of the Coulomb matrix are well established by now and are available in a number of electronic-structure codes. The only remaining problem in actual applications is that the crossover with the most efficient quadratic scaling algorithms is relatively late because the prefactor of the FMM method is relatively high.

There are many approximate algorithms for the construction of Coulomb matrices and a full review of all of them is outside the scope of this article. The arguably most successful quadratically scaling approximation to the Coulomb matrix is based on the resolution of the identity (RI) or density fitting (DF) approximation.^29−33^ To the best of our knowledge, the RI-approximation was conceptualized by Whitten^29^ and was used in its earliest form in one of the first molecular implementations of DFT by Baerends and collaborators.^30,31^ At this time, an atom-centered fitting basis set was introduced and the expansion coefficients were determined to maximize the overlap of the fitted density with the approximated density while constraining the fit to reproduce the integrated total number of electrons. In 1993 Vahtras and Almlöf studied various ways to determine the fit coefficients and concluded that the Coulomb metric is by far the best choice when the goal is to approximate Coulomb potentials.^33^ Dunlap pointed out that this way of determining the fit coefficients is effectively fitting the electric fields created by a charge distribution and he also discussed the variational nature of the fitting and the concept of robust fits that do not have a first-order error.^32,34^ Ahlrichs and co-workers then made important contributions to the practical use of the RI approximation in quantum chemistry by developing high-quality, compact fitting basis sets^35−38^ and provided an efficient implementation of the RI approximation to Coulomb potentials in the framework of DFT (RI-J). The results demonstrated that the RI-J approximation speeds up Coulomb matrix formations by a factor between 10 and 100.^35,36^ The reason for these impressive speedups are that the three-index electron repulsion integrals that need to be calculated twice in each SCF iteration are far less numerous and far less expensive than the four-index integrals occurring in a traditional Coulomb matrix calculation (Full-J). While the absolute errors introduced by the RI-J approximation are not small (as will also be discussed later in this article), error cancellation is spectacularly good. The remaining errors tend to be orders of magnitude smaller than basis set and method errors such that the RI-J and Full-J results can be used interchangeably for energy differences.

In 1999 Ahmadi and Almlöf^39^ used the McMurchie-Davidson (MD) integral formula^40^ to show that Full-J calculations can be sped up significantly by first transforming the density into a basis of Hermite Gaussians, then forming the Coulomb matrix in the Hermite basis and transforming it back to spherical harmonics Gaussian basis functions.^39^ The same method was rediscovered several times later and was given alternative names, such as the “J-engine.”^41,42^

In 2003 the same concept was used to propose the “Split-RI-J” method that shares with the original Ahmadi and Almölf proposal that the time-critical steps are carried out in the Hermite basis.^43^ The implementations showed that the Split-RI-J method can outperform traditional RI-J methods by up to a factor of 4. The method was reimplemented in an even more efficient way in 2021 concomitant with the introduction of the SHARK integral algorithm.^44^ To the best of our knowledge, the Split-RI-J algorithm is the most efficient of the quadratic scaling RI-based algorithms proposed to date and is widely used to form Coulomb matrices of all kinds in the ORCA package.^45,46^

The combination of RI/DF with multipole techniques has first been explored by Ahlrichs and co-workers as early as 2003.^24^ Their method, called “multipole accelerated RI-J” (MARI-J), was reported to lead to considerable savings for large molecules.^24^ However, since there was no hierarchical boxing algorithm used, linear-scaling was not reached. Yet, the algorithm achieves subquadratic scaling using carefully constructed thresholding techniques. It was commented that the extension of the algorithm to hierarchical multipoles would be straightforward. Sierka and co-workers subsequently reported a linear-scaling RI-based Coulomb algorithm for periodic systems.^26,47−49^ In a more recent paper, Csóka and Kállay reported the development of a multipole-based algorithm for the RI-based calculation of both, the exact exchange and the Coulomb terms.^50^ While the accuracy of the reported results is impressive, they appear to also not have used any kind of hierarchical boxing algorithm that would bring the scaling down into the linear regime.

In this paper, we report the development of a linear-scaling method for the formation of Coulomb matrices that is based on a new proposal of how to employ the multipole expansion. The method, called “Bubblepole” (BUPO) approximation shares similarities with the FMM method but differs significantly in key construction principles. The method can, in principle, be combined with the Full-J method to arrive at the BUPO-J algorithm that we have implemented but not extensively explored. Rather, we report here the combination of the BUPO approximation with Split-RI-J which leads to an algorithm that we refer to as RI-BUPO-J. We will first describe the theory and implementation before analyzing the achievable accuracy and demonstrating the computational efficiency.

## 2 Theory and Implementation

### 2.1 RI-J method

In the RI-J approximation a product of basis functions μ and ν from an atomic orbital (AO) basis set {μ} is expanded in terms of an atom-centered set of auxiliary functions {*K*}. The error Δ^μν^ of such an expansion for a given product of basis functions is
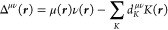
2

According to Almlöf and co-workers, the expansion coefficients *d*_*K*_^μν^ are best determined from minimizing the self-repulsion of the error. The residual Ω that is to be minimized is
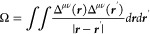
3

The condition  immediately leads to a linear equation system
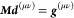
4With the positive definite metric ***M*** being given by the matrix of two-index repulsion integrals
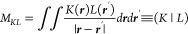
5and the right-hand side by three-index repulsion integrals
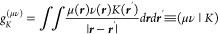
6

The linear equation system (**4**) is usually solved via the Cholesky decomposition of the metric matrix *M***.** This process is highly efficient and numerically stable. In practice, it is advantageous to employ a pivoted Cholesky algorithm since it is numerically more stable in the rare cases where the metric is nearly singular due to a (nearly) overcomplete fitting basis.

A Coulomb type matrix can be written as
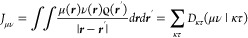
7with four-index repulsion integrals
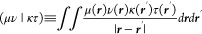
8***D*** is some density (or density like) matrix and ϱ(***r***) = ∑_κτ_*D*_κτ_κ(***r***)τ(***r***) is the associated electron density in real space.

Inserting the RI approximation into 7 results in a three-step procedure for the formation of an approximate Coulomb matrix. In the first step, the auxiliary vector ***g*** is formed which might be viewed as a projection of the density onto the space spanned by the auxiliary basis functions:
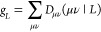
9

In the second step, the linear equations are solved in order to obtain the auxiliary-basis density:
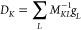
10where *D*_*K*_ is the fitted density in the auxiliary basis set. Finally, the RI-J approximation to the Coulomb matrix is formed:
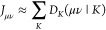
11

The algorithm requires the three-index integrals to be calculated twice in each SCF iteration. Since the number of significant μν products is linear-scaling with system size, the overall asymptotic scaling of the algorithm is *O*(*N*^2^) because the two-electron repulsion integrals decay as the inverse of the distance. The Cholesky decomposition formally has a cubic scaling with system size but its prefactor is so small that the time taken for the solution is negligible for all presently realistically approachable system sizes (vide infra). Linear-scaling variants of the Cholesky decomposition are known.^51^

### 2.2 Split-RI-J method

The Split-RI-J approximation makes use of the expansion of basis function products in terms of Hermite polynomials by means of the McMurchie–Davidson^40^ construction:^43^
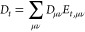
12where “*t*” is a generic compound label signifying Hermite polynomials. The E-coefficients are readily generated by recursion.^40^ Within the SHARK-algorithm,^44^ they are conveniently represented as a matrix *E*(*t*, μν), where μν refers to the basis function products within one shell pair. Please note that the expansion 12 is exact.

The next step consists of forming the auxiliary vector *g*_*t*′_ in the Hermite basis:
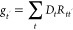
13

Followed by back transformation into the spherical harmonics Gaussian basis resulting in the auxiliary vector *g*_*K*_ that is identical to the one in eq 9.
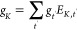
14Here *E*_*K*,*t*′_ are the Hermite expansion coefficients for the auxiliary basis set. After solving the linear eqs 4 for the auxiliary-basis density, a similar sequence leads to the formation of the RI-J Coulomb matrix. First, one forms the auxiliary density in the Hermite basis:
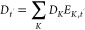
15

Followed by the Hermite basis RI-J Coulomb matrix *J*_*t*_:
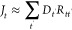
16Here *R*_*tt*′_ is the repulsion integral over two Hermite Gaussians. Details on how to calculate these integrals can be found in the original publications^40,43^ as well as the publication describing the SHARK algorithm.^44^

The final step is the back transformation into the spherical harmonics Gaussian basis:
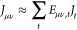
17

The Split-RI-J method leads to the exact same results as the traditional RI-J method. The rate-limiting steps, namely the formation of the Hermite basis auxiliary vector and the Hermite basis Coulomb matrix are, however, computationally significantly faster than in the RI-J method because the R-coefficients can be computed with high efficiency and no overhead from unnecessary transformations, such as the one arising from Cartesian Gaussians to spherical harmonics (the latter transformation is folded into the definition of the E-coefficients that are precomputed^44^). While the scaling of the algorithm is identical to the scaling of the RI-J algorithm, the prefactor of the Split-RI-J algorithm is smaller by a factor of up to 4.^43^ As analyzed in the original publication,^43^ the advantages of Split-RI-J over RI-J increase for larger and more accurate basis sets with higher angular momentum functions.

### 2.3 Multipole Expansion of Two-Electron Integrals

If the two charge distributions on the bra- and ket-side of a given electron–electron repulsion integral do not overlap, the integrals 5 and 8 can be approximated to high precision by a multipole approximation. In this work, we only approximate the three-index repulsion integrals 6. We closely follow the notation of ref (28) and emphasize that we include this exposition here only in order for the article to be self-contained. We have carefully repeated the derivations presented in ref (28) and have not found a single mistake or misprint.

Let us write the charge distributions on the bra and ket sides as ϱ_*P*_(***r***) and ϱ_*Q*_(***r*** ′) respectively and let ***P*** and ***Q*** be the chosen centers for the multipole expansion. We then have
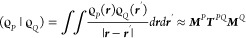
18where ***M****^P^*are the multipole moments of charge distribution ϱ_*X*_ (X = *P*, *Q*) and ***T****^PQ^* is the multipole interaction tensor. The multipole moments are given by
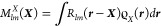
19

The *R*_*lm*_ are scaled-solid spherical harmonics that are related to standard spherical harmonics by^28^
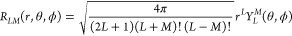
20

The expansion on both, ϱ_*P*_ and ϱ_*Q*_ is taken to a given maximum angular momentum *L*_max_^(*X*)^ which defines the length of the multipole vector ***M****^X^*. In our implementation arbitrary-order multipole integrals are first calculated over Cartesian multipoles using a recursive algorithm^28,52,53^ (summarized in the appendix) followed by a transformation to spherical harmonics.

The multipole interaction matrix ***T****^PQ^* only depends on the distance and relative orientation (e.g., the difference vector ***P–Q***) of the two charge distributions and is given by
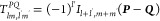
21

The scaled irregular solid spherical harmonics *I*_*L*, *M*_ are related to standard spherical harmonics by
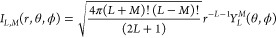
22

The multipoles *M*_*lm*_^*X*^(***X***) calculated at a given expansion point ***X*** can be translated to another point ***Y*** according to
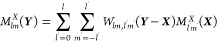
23

The translation matrix *W*_*lm*, *l* ′ *m*′_ is simply:
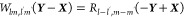
24

It is important to note that, in general, an expansion that is exactly terminating at a given value of *l* at point ***X*** is no longer exactly terminating at any finite angular momentum value at another expansion point ***Y***. The matrix ***W***, by construction, is a lower triangular matrix with unit diagonal. It can be used to “up-translate” a given multipole expansion by choosing a *l* > *l*′. This will become significant for the construction of our algorithm below. During the course of this work, we observed that truncating a nonexact multipole expansion at a specific angular momentum *l* results in larger errors in the approximated integrals as the multipoles are translated further from their original expansion center. In order to compensate for these increased errors, we found it customary to increase the expansion length once multipoles are translated to higher levels of the multipole hierarchy. We refer to this as “up-translation”.

Using the definitions 20 and 22, the multipole moments 19 as well as the translation 24 and interaction 21 matrices are complex valued. However, it is unnecessary to employ complex algebra since the final quantity, the multipole expansion integrals 18, are real-valued. As explained in detail in ref (28) one can rewrite the entire expansion in terms of real quantities of the same dimensions (eqs 9.13.49, 9.13.58–61 and 9.13.71–74 in ref (28)). Effectively this means, that the integral approximations ***M****^P^****T****^PQ^****M****^Q^* can be formed by two matrix multiplications in real algebra instead of effectively six matrix multiplications that would be necessary in the complex case.

At the request of a referee, we make this statement explicit by considering a triple matrix product of the form ***Z = XYX***^′^, where ***X,Y*** and ***X***′ are complex matrices but we know that ***Z*** is real-valued. Let us write ***X*** = ***A*** + *iB***,***Y* = ***C*** + *i****D*** and ***X*** ′ = ***A*** ′ + *i****B***′ (where ***A,A***′***,B,B,C,D*** are real-valued). One readily sees that the formation of ***Z***
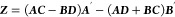
25

Requires six matrix multiplications of real-valued matrices of dimension *N* leading to a computational complexity of *O*(6*N*^3^). If, however, we can express ***X,Y,X***′ in a way that they are real-valued and still have dimension *N*, then the computational complexity to form ***Z*** is only *O*(2*N*^3^) leading to savings of a factor of 3. This is the case for the multipole integrals ***M****^P^****T****^PQ^****M****^Q^* with the explicit equations for the real-valued ***M****^X^* and ***T****^XY^* (X,Y = P,Q) given in ref (28). In our implementation we therefore use real algebra throughout and also avoid the unnecessary computation of the complex valued *R*_*LM*_ and *I*_*LM*_.

### 2.4 Exact versus Approximate Expansions

If the multipole expansion is performed at the “natural center” of an auxiliary shell or a shell pair, the expansion will exactly terminate at a finite value of *l*. This is a desirable feature since exact expansions avoid any truncation error and are very short, thus leading to computational savings.

The following expansions are exact and terminating:

1. Auxiliary shells expanded at their parent atom. For a shell of angular momentum *l*_*K*_, the expansion terminates exactly at *l*_*K*_. The multipoles with *l* < *l*_*K*_ are also equal to zero but exploiting this feature does not appear to be worthwhile from a computational point of view.
2. The expansion for a primitive Gaussian shell pair with angular momenta *l*_μ_ and *l*_ν_ expanded at its Gaussian product point (where *a*, *b* are the primitive Gaussian exponents of the Gaussians centered at ***R****_A_* and ***R****_B_*, respectively) terminates exactly at the sum of the angular momenta *l*_μ_ + *l*_ν_ of the two Gaussians.
3. The expansion for a contracted shell pair with both Gaussians located at the same atom exactly terminates at the sum of the angular momenta of the two Gaussians.

A contracted shell pair with more than one primitive shell pair will not terminate exactly and will therefore require a longer multipole expansion in order to reach full accuracy. Alternatively, each primitive pair of a contracted shell pair can be expanded separately at its Gaussian product point, thus leading to an exact and terminating expansion again at the expense of generating a much larger number of multipoles.

In our RI-based multipole algorithms described below, we will ensure that either the bra- or the ket-side of the multipole expansion is an exact expansion. This greatly helps to keep the required multipole expansions short and the accuracy of the approximation high. This construction also keeps the prefactor of the multipole-based part of the calculation small since it is neither necessary to generate high-order multipole integrals nor is it necessary to multiply high-dimensional matrices to obtain the actual multipole approximations in eq 18.

### 2.5 Shell Pair and Auxiliary Shell Extents

The multipole expansion is only permissible if the two charge distributions do not overlap. This is trivial to ensure for point charge distributions. However, for the continuous charge distributions met in quantum chemistry, it is necessary to define an “extent” of a charge distribution in order to arrive at a multipole allowedness criterion. In their original work, White and Head-Gordon proposed an extent (*ext*_α, β_) definition for a primitive pair of basis functions with Gaussian exponents α and β:^18^
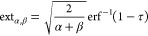
26Here τ is an accuracy parameter that is usually reported to be chosen in the range 10^–4^–10^–6^. This equation can be derived for a pair of s-functions but is apparently used for shell pairs of any angular momentum in most existing FMM implementations.

For a contracted shell pair, the situation is more complicated. A contracted Gaussian function with angular momentum *l*, centered at atom A, may be written as
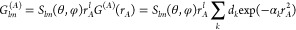
27with ***r****_A_* = ***r***–***R****_A_*, *r*_*A*_ = |***r****_A_*|, and ***R****_A_* being the position of atom A. For each pair of primitives centered on atoms A and B respectively, the GPT states that the resulting Gaussian is centered at a point ***P***_αβ_
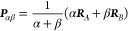
28

**Figure 1:**
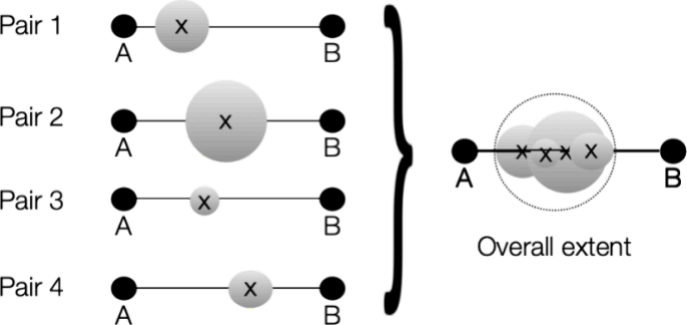
Relationship of the individual shell pair centers and extents to the contracted shell pair center and extent.

Hollman et al.^54^ suggested to calculate the expansion center for a contracted pair as a weighted average of the individual primitive expansion centers (Figure 1). Writing the two contracted Gaussians centered at points A and B respectively as *G*_*I*_^(*A*)^, *G*_*J*_^(*B*)^, the definition of the expansion center is
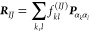
29where the sums *k*, *l* are over the primitives of the two Gaussians. The weighting factors *f*_*kl*_^(*IJ*)^ are defined as
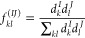
30

The extent of the contracted charge distribution is then calculated as
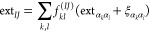
31

The quantity ξ_α_k_α_l__ takes account of the fact that the individual primitive centers ***P***_α_*k*_α_*l*__ are displaced from the global shell pair center ***P****_IJ_* (Figure 1):
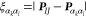


In our opinion, these definitions are plausible and safe, but have at least two significant problems:

1. The extent definition is only valid for s-type Gaussians. The true radial dependence is *r*_*A*_^*l*^*G*^(*A*)^(*r*_*A*_) and not *G*^(*A*)^(*r*_*A*_). Leaving out the factor *r*_*A*_^*l*^ leads to an incorrect description of the radial behavior of the Gaussian shell.
2. The extent definition for a contracted pair will tend to make the extents larger and larger as the distance between the two atoms increases due to the factors ξ_α_k_α_l__. However, at the same time, the overlap of the two Gaussian shells gets smaller and smaller. Hence the true extent of the Gaussian shell pair is *shrinking* with interatomic distance while the extent of the contracted shell pair, according to eq 31, is *increasing*. A similar problem appears to be present in the extents used in refs (24) and (50). We also note a careful discussion and derivation of an alternative extent by Kudin and Scuseria in ref (27).

In order to address these two issues, we propose a new definition for the extent of a contracted or primitive shell pair. This is a highly important subject since the shell pair extents and their reliability are critically important for a reliable near-field/far-field separation later in the algorithm. In our definition of the shell pair extent, we retain the definition of the contracted shell pair center, eq 29, as a reasonable compromise for a common expansion point. However, we choose a different approach to the definition of the shell pair extent. It is first and foremost important to realize that the shape of the spherically averaged Gaussian shell pair
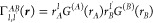
32is fairly complicated and may show a number of local minima. We are, however, only interested in the radius of the sphere that encloses Γ_*l*, *l′*_^*AB*^(***r***), such that outside the sphere Γ_*l*, *l′*_^*AB*^(***r***) is certainly below a predefined threshold *T*_sphere_. In order to determine this radius (that will be taken as the extent of the shell pair), we first calculate the same sphere radii *S*(*I*^(*A*)^) that are used in the chain of spheres exchange (COSX) algorithm.^12^ The *S*(*I*^(*A*)^) define a sphere around Gaussian shell *G*_*I*_^(*A*)^ outside of which *G*_*I*_^(*A*)^ is guaranteed to be < *T*_sphere_. This allows us to choose a “safe” distance at which the shell (and therefore also the shell pair product) will be negligible. We then put two virtual “walkers” along the connecting line between the two shell pairs and scan inward in increments of 0.01 Bohr until we meet the points *z* = *z*_max_ and *z* = *z*_min_ where |Γ_*l*, *l′*_^*AB*^(***r***)| > *T*_sphere_. Their distance from the shell pair center *C* then defines the extent of the shell pair as
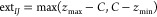
33

**Figure 2:**
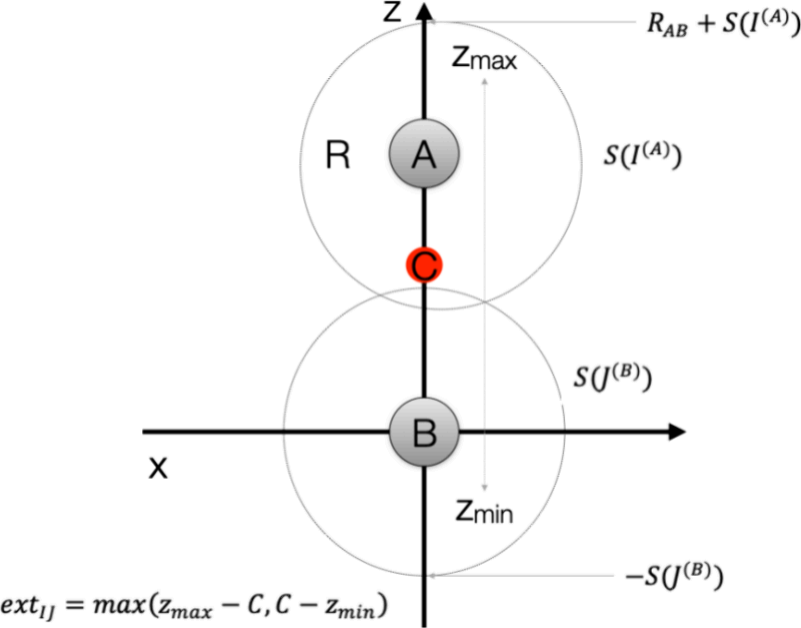
Geometric construction used for the definition of the shell pair extents in this work. Gaussian B is placed at the center of the coordinate system and Gaussian shell A at the interatomic distance ***R*_*AB*_** along the *z*-axis. The scan leads inward in from *R*_*AB*_*+ S*(***I***^(***A***)^) in -z direction and from **–***S*(***I***^(***B***)^ in +z direction until the points ***z*_max_*,z*_min_** are found for which |**Γ**_***l,l′***_^***AB***^(***r***)| ***> T*_sphere_** is satisfied. The distance of these points from the shell pair center ***C***, that is necessarily located somewhere between 0 and ***R*_*AB*_** then defines the shell pair extent.

The geometry of the construction is most easily understood with respect to Figure 2.

This construction is safe and ensures that the product of the two Gaussian shells is negligible to within *T*_sphere_ everywhere in space. As one final safety measure, extents smaller than 2 Bohr are not allowed. This is not strictly necessary but avoids possible underflows when very tight core-orbitals are involved. The extents defined in this way properly shrink with increasing interatomic distance and, in our opinion, are as tight as possible. A typical average shell pair extent for a standard basis set, such as the def2- bases,^38^ is 5–7 Bohrs, whereas it would be 2 or 3 times larger with the literature definition. For an auxiliary shell *K*, the extent is simply defined as *S*(*K*^(*A*)^), which is rigorous.

### 2.6 Near-Field/Far-Field Separation

Given the extent definitions, we can now define the near-field/far-field (NF/FF) separation. The multipole approximation for two charge distributions *P* and *Q* is valid if the following criterion is met:
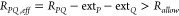
34

As discussed by Lambrecht and Ochsenfeld,^17^ a value of *R*_allow_ ≥ 1 guarantees that the powers *R*^–*L*–*L*′–1^ in the interaction matrix become smaller for increasing expansion lengths, thus ensuring a convergent multipole expansion. We have experimented extensively with different value of *R*_allow_ and have settled on *R*_allow_ = 1 to be perfectly acceptable while ensuring the largest possible size of the FF.

### 2.7 Recursive “Bubble” Hierarchies

In addition to the definition of the extents, we chose a fundamentally different approach to the overall construction of the near- and far fields than algorithms that use boxes. Our construction uses spheres that enclose a given number of objects (shell pairs, auxiliary shells or point charges). We refer to these spheres as “bubbles”. These bubbles are constructed such that they “host” a number of objects that are spatially close. The radii of the bubbles are chosen such that they enclose all extents as well. Thus, it is guaranteed that outside the boundaries of each bubble no shell pair or auxiliary shell can have a value >*T*_sphere_. This eliminates all uncertainties or complexities arising from “leakage” out of box boundaries once and for all.

Our bubble construction algorithm is based on the popular “Kmeans” clustering algorithm.^55,56^ Given a list of object centers ***C*** and extents ***E***, the algorithm finds clusters of objects in close spatial proximity in a way that each bubble hosts approximately the same number of objects (*TargetDim*). Thus, the bubbles adapt themselves perfectly to the shape of the molecular system under investigation. We will numerically investigate the best choice for the target dimension below but will mention in passing that values around 150 shell pairs appear to be appropriate. Given that a large molecule has several hundred thousand significant shell pairs, this means that the number of bottom-level bubbles is also in the thousands.

**Figure 3:**
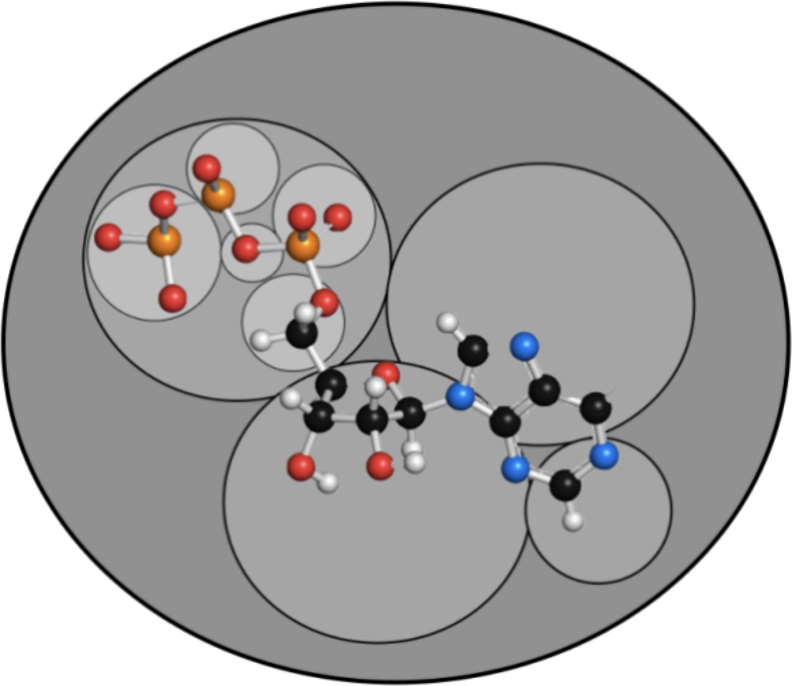
Illustration of the recursive bubble hierarchy that orders objects (shell pairs, auxiliary shells or point charges) into spheres or “bubbles” that enclose an approximately equal number of objects. Subsequently, bubbles are combined into superbubbles in an iterative manner (levels) until the entire molecular system is enclosed in one “top-level” bubble.

Following the construction of the bottom-level bubbles that enclose the actual objects, the algorithm proceeds to combine several bubbles into one superbubble of the next level. The process is repeated until the entire molecular system is enclosed in a single top-level bubble. The number of bubbles that go into a superbubble is user definable (*TargetDim*2). However, we found a value of *TargetDim*2 = 3 to be appropriate for all circumstances that we have met so far. The construction is illustrated in Figure 3.

Following the construction of the bottom-level bubbles, the algorithm proceeds by calculating the bottom-level multipole expansions of a certain length (discussed below) at the respective bubble center. The bottom-level multipoles are then recursively up-translated to the centers of the superbubbles where the angular momentum of the expansion is increased in each step until either the top-level bubble is reached or a predefined maximum angular momentum (around 40) is reached. We refer to these multipoles enclosed in bubbles as “Bubblepoles” and consequently give the algorithm the acronym “BUPO.”

**Figure 4:**
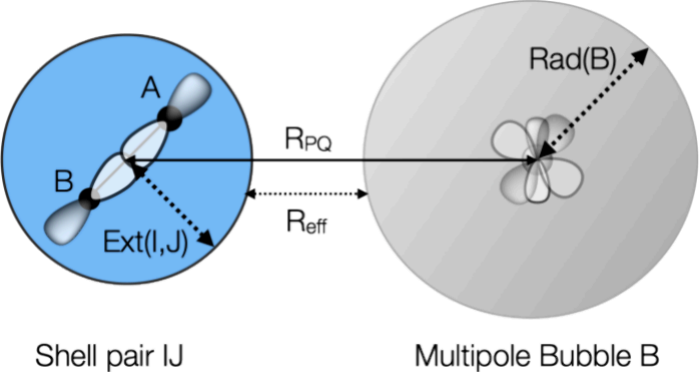
Visualization of the near-field/far-field separation in the BUPO algorithm. On the bra side there is an object (shell pair, auxiliary shell or point charge) defined by its center and extent. It is interacting with the multipoles of a given bubble defined by the bubble center and radius. The effective distance used to determine whether the multipole approximation is allowed is given by the distance of the object and bubble centers minus the sum of the extent and the bubble radius.

The NF/FF separation then can be visualized as in Figure 4. The definition of the extents and bubbles lends itself to a very clean, efficient and natural NF/FF separation.

Proceeding in this way, it becomes evident, how the algorithm is “carving out” the far field for a given target (bra) object. A given object located in a given part of the molecule may find the multipole criteria to be satisfied for a very large, distant bubble at which point two simple matrix multiplications will take care of all interactions that the target object has with the distant part of the molecule. What we are implying with this statement is that the interaction of a given target (‘P’) with the entire distant part of the molecule (‘Q’) only requires a single evaluation of the triple matrix product ***M****^P^****T****^PQ^****M****^Q^* where in a traditional algorithm millions of prescreening operations and integral evaluations would be required. The matrices ***M****^P^*, ***T****^PQ^*, ***M****^Q^* still need to be evaluated of course.

**Figure 5:**
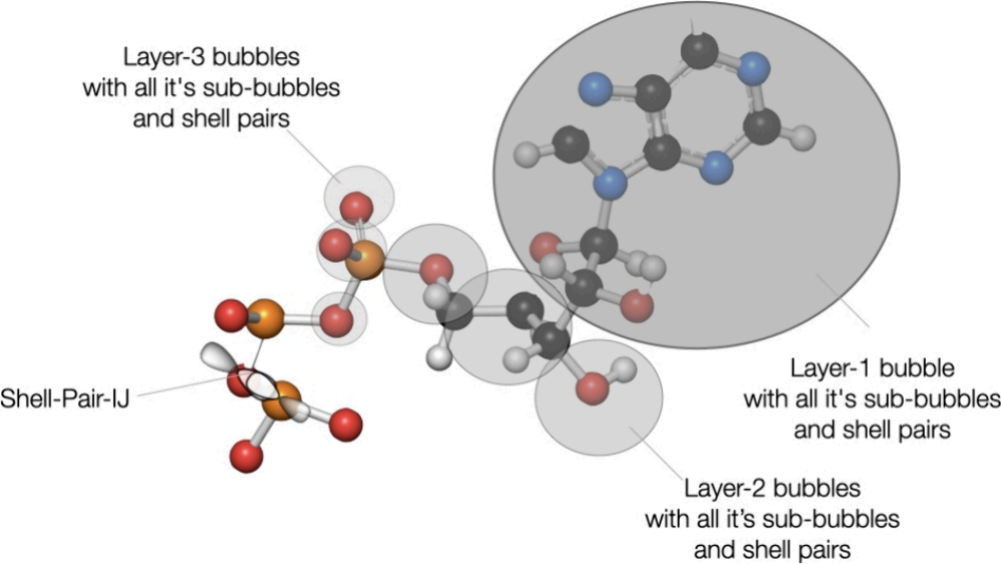
Graphical illustration of how the BUPO algorithm carves out the near field for a given target object. At the top level it may find a very large bubble that encloses most of the distant parts of the molecule. The interaction with this part of the molecule can then be calculated with a single multipole interaction (two matrix multiplications). Beyond that top-level bubble, the bubbles at lower levels become smaller and smaller and enclose smaller and smaller parts of the molecular topology. The total number of bubbles at any level that meet the far-field criteria define a “far-field surface”.

Proceeding down the hierarchy of bubbles, the bubbles become smaller and smaller and enclose smaller and smaller parts of the molecule. The entirety of bubbles that meet the far-field criteria for a given target object then define a kind of “far-field surface” that is closely following the topology of the molecular structure. This is illustrated in Figure 5.

The presence of diffuse basis functions often plagues the application of multipole-based algorithms. In our implementation this problem will be dealt with by separating out diffuse shells and shell pairs and placing them in an extra “throwaway bubble” that will always be in the near field. In this way, the presence of diffuse basis functions does not compromise the exploitation of locality of the nondiffuse shells. It should be noted, however, that diffuse functions will break the linear scaling of the algorithm to some extent. We should note, however, that in our studies, we tend to avoid the use of superfluous diffuse basis functions because, in large molecules, they will cause linear dependencies in the basis set from which there is no convincing recovery.

## 3 BUPO-J Algorithm for Coulomb Matrices

### 3.1 Outline of the Full BUPO-J Algorithm for Coulomb Matrices

The BUPO construction can be used in many contexts. In this paper, we restrict ourselves to the calculation of approximate Coulomb matrices and will report further developments in due course.

**Scheme 1:**
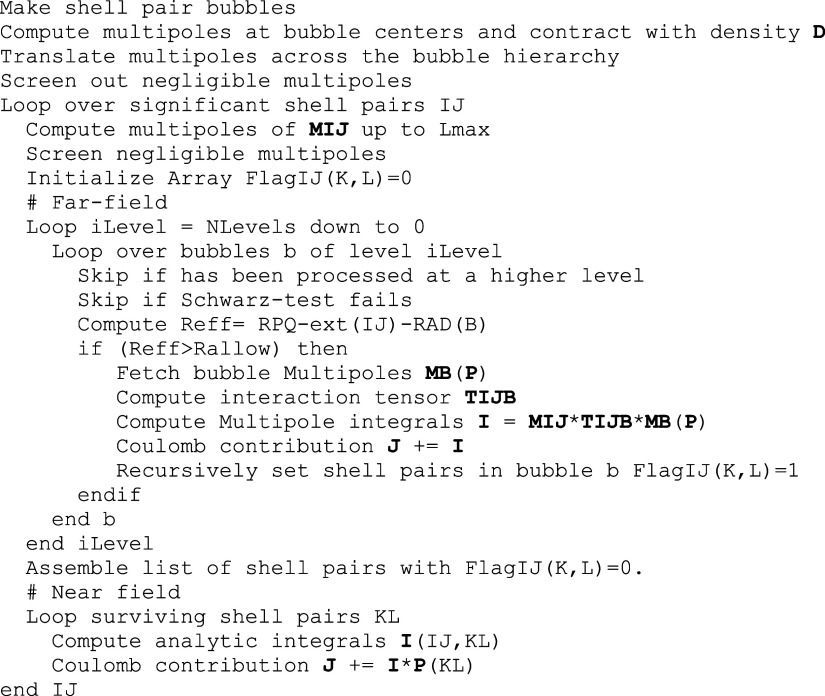
Pseudo-Code for a BUPO-Based Calculation of a Coulomb Matrix

The construction of a BUPO-based algorithm can perhaps be most readily understood with respect to the pseudocode in Scheme 1. The algorithm commences with the construction of the shell pair bubbles. It is not necessary to perform this step in each SCF iteration and consequently, this is only done once at the startup of the run. The step is, however, so cheap that it could be repeated without compromising efficiency (see Section 4.8). The next step is the calculation of the bottom-level bubble multipoles and their contraction with the density (or difference density) matrix followed by translation of the multipoles across the bubble hierarchy. This step can be followed by a removal of the negligible multipoles at each level according to a screening parameter *T*_screen_. The optimal value of this parameter will be discussed in the numerical section. These aforementioned preparatory steps are the bulk of the overhead that the BUPO algorithm produces relative to a fully analytic calculation.

The “productive” part of the algorithms is driven by an outer loop over non-negligible shell pairs. For a given shell pair, the multipoles are computed again at its natural expansion center. For a contracted shell pair, the options exist to either choose the contracted shell pair center or the primitives centers of each surviving primitive pair. In the latter case, the expansion terminates exactly at *l*_*i*_ + *l*_*j*_ while in the former case the expansion is nonterminating and needs to be carried to a predetermined maximum angular momentum *L*_*max*_ possibly followed by a screening step.

The next step consists of the far-field calculation. Here, there is an outer loop over bubble levels. At each level there is a loop over the bubbles that exist at this level. It is advantageous to introduce a Schwarz prescreening step here. For each bubble, we store a value of *PKMAX* = max_*I*, *J* ∈ bubble_(*K*_*IJ*_*D*_*IJ*_) where for two shells *I*, *J* the values are  and *D*_*IJ*_ = max_*i* ∈, *j* ∈ *J*_(|*D*_*ij*_|). If more than one density is processed, the maximum values are taken over all densities. These values can then be used to perform a Schwarz prescreening test by skipping the contribution if  (with *Thresh* being the default neglect threshold for the direct SCF procedure). This screening is helpful in particular in the later SCF cycles where the difference density (that is used in place of the density for incremental Fock matrix builds^57,58^) is getting small. If a given bubble survives the Schwarz-prescreening, the multipole criterion is evaluated. If the multipole approximation is allowed, the multipole interaction between the shell pair and the bubble multipoles is computed and the Coulomb matrix is updated. In order to avoid redundant computation, a list with near-field shell pairs (those not treated in the far-field part of the calculation) is assembled on the fly. This procedure is carried through to the bottom level of the hierarchy. The net result of this step is the far-field Coulomb matrix as well as a list of shell pairs that needs to be treated in the near field.

The near-field portion of the algorithm is then done in the traditional way with the one exception that only shell pairs that have not been treated in the far field will be processed. A standard Schwarz-prescreening test will be calculated and the product of the four-index integrals with the respective ket shell pair density matrix is added to the Coulomb matrix.

### 3.2 Outline of the RI-BUPO-J Algorithm for Coulomb Matrices

The very same principles described above are also followed in creating the Split-RI-J version of the BUPO-based Coulomb formation algorithm as shown in Scheme 1.In this scheme, the multipole approximation needs to be applied twice—once in the formation of the auxiliary vector and once in the completion of the RI-based Coulomb matrix. It will turn out that the requirements to reach sufficient accuracy will be quite different for the two steps as will be discussed in detail in the numerical section.

**Scheme 2:**
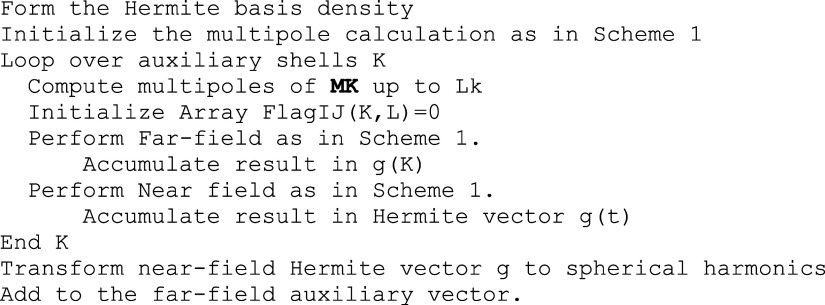
Pseudo-Code for the First Step of the RI-BUPO-J Calculation That Leads to the Auxiliary Vector g

A simplified pseudocode for the completion of the first step of the RI-BUPO-J algorithm is shown in Scheme 2. The initial steps of the calculation and the setup of the shell pair multipoles is identical to what was described for the parent BUPO-J algorithm in Scheme 1. The additional step in RI-BUPO-J is the transformation of the density into the Hermite basis that is computationally insignificant. The outer loop processes the auxiliary shells K. For each auxiliary shell, the multipoles are generated up to its own angular momentum Lk. As remarked in Section 2.4, the lower multipoles are obviously zero in this case, but it is computationally not worthwhile to explore this. The same shell pair flag array as in the BUPO-J case is initialized before entering the far-field section in which the interaction of the auxiliary-basis multipoles with the shell pair bubble multipoles is computed. The product of this part of the calculation are the shell pair flags as well as the far-field part of the auxiliary vector.

The solution of the linear equation system proceeds in the same way as for the standard RI-J or Split-RI-J algorithms. Again, this step is computationally insignificant. Should it lead to recognizable deviations from linear-scaling or create computational bottlenecks, it will be linearized. This was not found to be necessary so far and we provide an example for how insignificant that step is in Section 4.8.

**Scheme 3:**
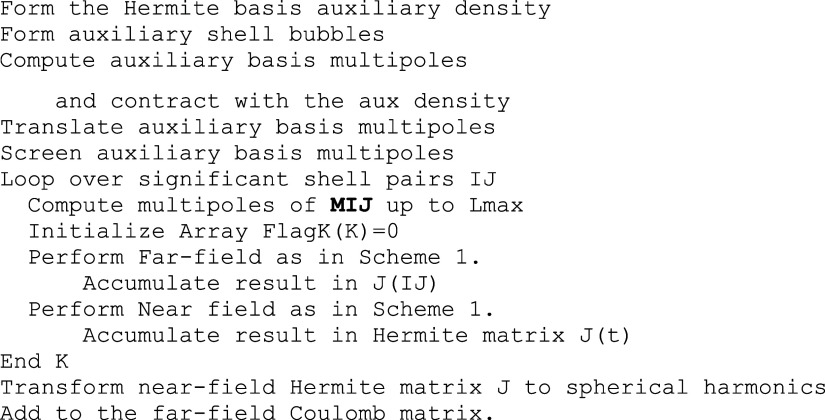
Pseudo-Code for the Final Step of the RI-BUPO-J Calculation That Leads to the Coulomb Matrix J

In the final step of the algorithm the Coulomb matrix is formed as in Scheme 3. In this step it is necessary to set up a second bubble hierarchy, this time for the shells of the auxiliary basis set. Here there are two options in the code: (1) we can set up the auxiliary shells as we would set up shell pairs by fixing a desired target dimension at the bottom level; (2) use “atomic bubbles”. In the latter case, all auxiliary shells of a given atom are combined in a given bubble. For this construction, it is guaranteed that the auxiliary shells all have the same expansion center that coincides with their parent atomic center. In this case, the bottom-level multipoles are terminated exactly at the maximum angular momentum that is included for the atom in question which leads to extremely short expansions. At the higher levels, the bubble centers do no longer coincide with atomic centers and up-translation is necessary.

The rest of the algorithm proceeds in close analogy with the first step. The calculation is driven by an outer loop over significant shell pairs. There are two options to generate the shell pair multipoles: (1) calculation of the multipoles at their contracted shell pair center. This expansion is not exact and therefore needs to be taken up to a predefined angular momentum Lmax. We will come back to this subject in the numerical part of the calculation. While the generation of higher-order multipoles and their subsequent processing creates some computational burden, it is also compact since all primitive shell pairs are treated with the same multipoles. (2) Generation of the shell pair multipoles at the primitive pair Gaussian product points. This expansion is exact, but requires the processing of a potentially large number of primitive pair multipoles which creates a certain amount of computational overhead.

In the far-field part, the calculation proceeds by evaluating the interaction of that shell pair with the auxiliary bubble hierarchy. Auxiliary shells that have been treated are flagged. The near-field calculation generates the three-index integrals exactly and adds the results of the contraction of the three-index integrals to the Hermite basis Coulomb matrix. After conclusion of the main loop over shell pairs, the Hermite basis Coulomb matrix is back transformed to spherical harmonics and added to the far-field Coulomb part.

## 4 Numerical Evaluation

In this section we will evaluate the accuracy and efficiency of the RI-BUPO-J algorithm. To this end, we will use a standard test system, namely linear glycine chains. These chains were constructed using the Avogadro molecular builder^59^ making sure that the construction would always enforce angles for an α helix structure. They were then further optimized using the GFN-FF force-field prior to running the benchmarks.^60^

Linear chains are suitable to demonstrate whether a given algorithm is linear-scaling or not and to establish its accuracy and performance. However, linear chains, of course, represent a “best-case” scenario for linear-scaling algorithms and are not necessarily indicative of what an algorithm is capable of in real-life applications. Therefore, we will also investigate a few more real-life systems that are closer to the true 3-dimensional systems one is likely to meet in practice. As always, we will first ensure sufficient accuracy in approximations before discussing the efficiency of the algorithm.

Before evaluating the accuracy of the multipole approximations themselves, the accuracy of the reference data should be investigated. Until otherwise noted, all calculations were done with the PBE functional,^61^ the def2-SVP basis set^38^ and the def2/J fitting basis set.^38^ The behavior of the algorithm for larger basis sets will be investigated after establishing the basic behavior of the algorithm.

**Figure 6:**
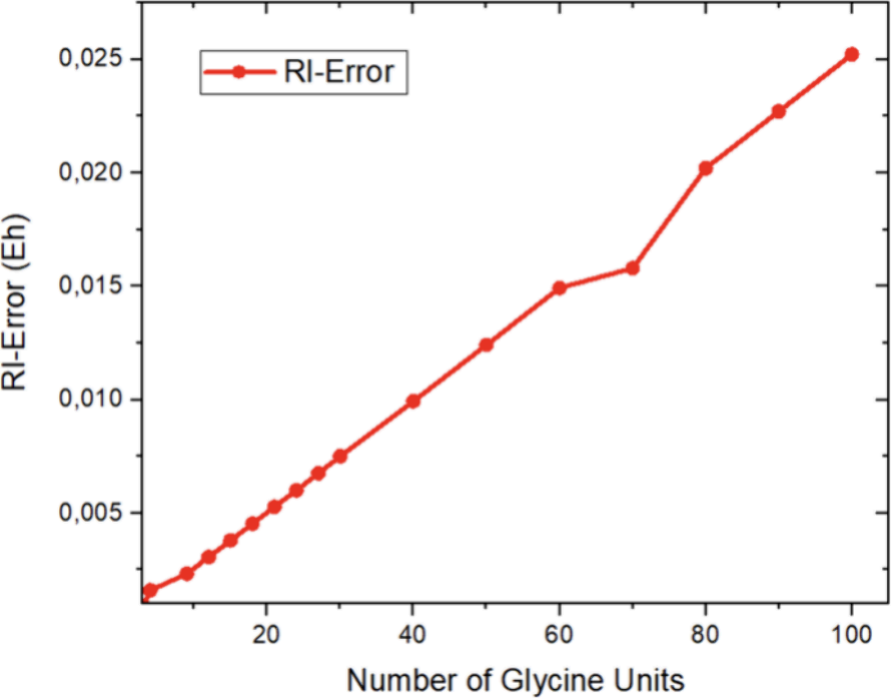
Error of the RI-J approximation calculated as the difference between a PBE/def-SVP calculation on the respective glycine chains with and without introduction of the RI-J approximation. The kink observed for (gly)_70_ is likely related to an irregularity in the structure and was not further investigated.

In order to better understand the error sources, we have first evaluated the error introduced by the RI-J approximation itself. The results in Figure 6 re-emphasize the well-known fact that the error introduced by the RI-J approximation is substantial and amounts to dozens of mEh for larger systems. However, these errors are highly systematic and extremely constant on a per-atom basis. This allows for a very high degree of error compensation which, together with its high efficiency, renders the algorithm so successful in practice.

**Figure 7:**
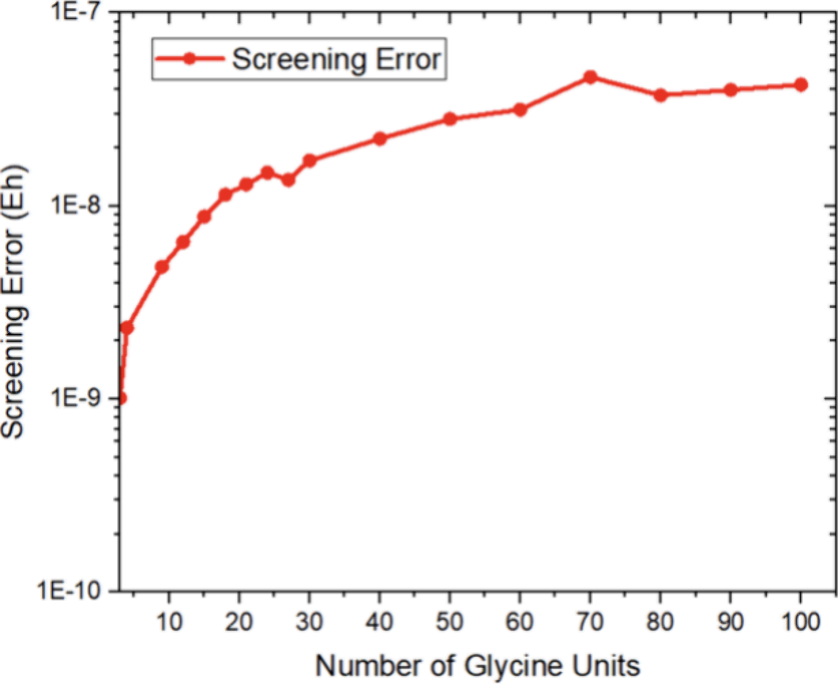
Accumulated numerical noise in the reference results calculated as the difference between “VeryTightSCF” and “ExtremeSCF” settings for RI-J-based PBE/def2-SVP calculations on a series of linear glycine chains.

Second, in calculations on large systems, it is important to worry about the accumulation of numerical noise. This is particularly important because traditional screening-based and multipole-based algorithms, that are based on presummation of small quantities, differ significantly in this respect. We have therefore compared the energies obtained with ORCA’s standard “VeryTightSCF” screening and convergence thresholds with calculations where all thresholds were set to 0 (ORCA’s “ExtremeSCF”). While the results in Figure 7 show the expected accumulation of numerical noise for larger glycine chains, it is gratifying to see that even for (gly)_100_ with more than 800 atoms, the results are still accurate to within 2–3 × 10^–8^ Eh. Thus, the numerical precision of reference results can be safely trusted to be within this accuracy.

### 4.1 Accuracy in Step 1

In this part of the investigation, we have performed the first step of the RI-BUPO-J calculation with the multipole approximations while performing the second step exactly as in the fully analytic Split-RI-J calculation. Since only the comparison to the standard Split-RI-J algorithm matters, these comparisons were done using a single Coulomb matrix build using the initial guess density provided by ORCA (“PModel” guess in ORCA). We will later demonstrate that the actually converged SCF results show the same kind of accuracy. All results were obtained for (gly)_50_.

**Figure 8:**
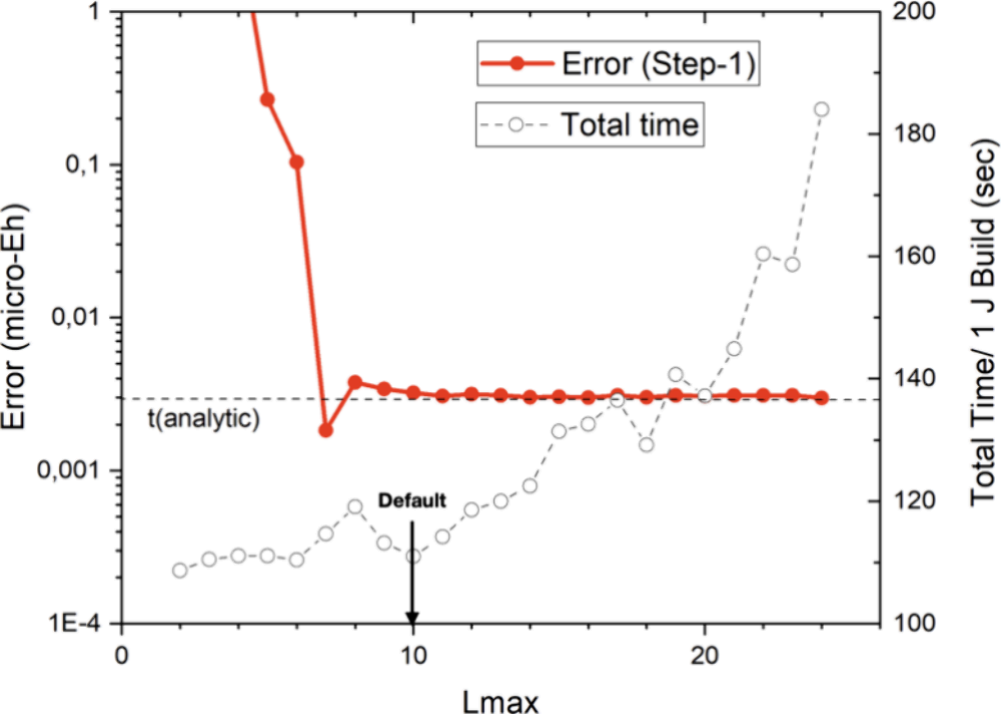
Convergence of the RI-BUPO-J method with respect to the expansion length of the shell pair multipoles in the shell pair bubbles. The red dots represent the error introduced by the multipole approximation, while the open circles represent the time required for one Coulomb build with the RI-BUPO-J algorithm. The horizontal dashed line is the time taken by the standard Split-RI-J algorithm. All results for (gly)_50_ were obtained with the PBE functional, def2-SVP basis set and def2-J auxiliary basis set.

We first investigate the convergence of the results with respect to the expansion length of the shell pair multipoles within the shell pair bubbles (*Lmax*). The results in Figure 8 demonstrate that the energy is converging very quickly to 10^–8^ Eh accuracy, the numerical limit of the reference results. We have chosen *Lmax* = 10 as our default value. This is a conservative choice and acceptable accuracy will also be obtained with smaller *Lmax* values as discussed below. We also show the dependence of the time required for a single Coulomb build in Figure 8. The results show, that up to about *Lmax* = 12, the total time is largely unaffected by the choice of *Lmax*. Only for long expansion length of, say, *Lmax* > 15 do the calculations become noticeably more expensive. The results in Figure 8 also demonstrate that actual computational savings over the highly efficient Split-RI-J algorithm are possible.

**Figure 9:**
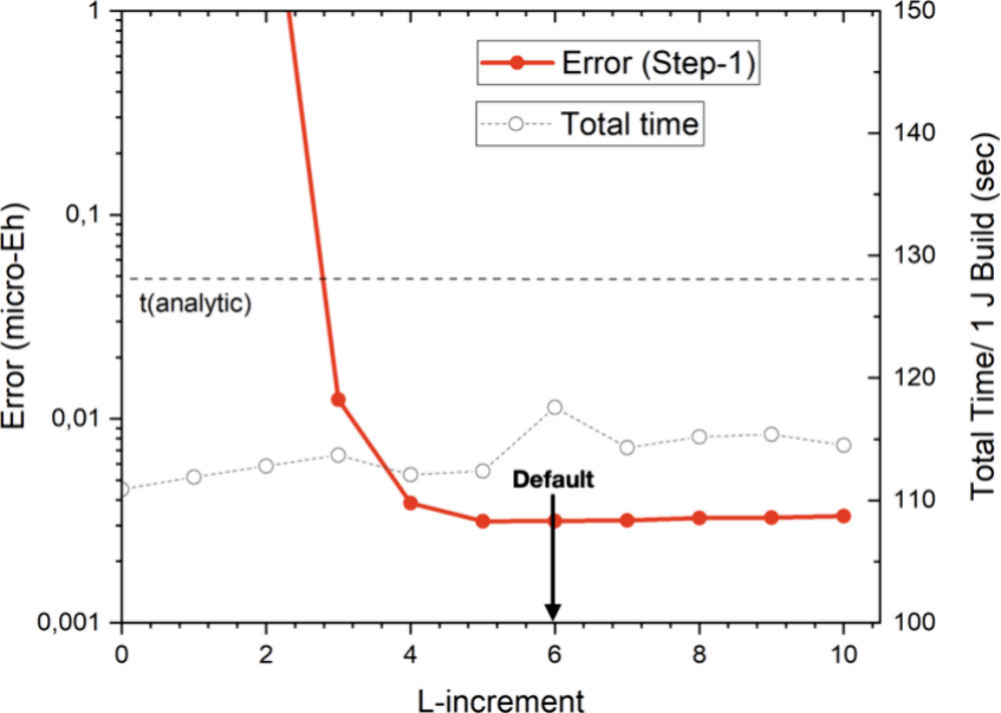
Convergence of the RI-BUPO-J method with respect to the up-translation parameter Lincr. The red dots represent the error introduced by the multipole approximation, while the open circles represent the time required for one Coulomb build with the RI-BUPO-J algorithm. The horizontal dashed line is the time taken by the standard Split-RI-J algorithm. All results for (gly)_50_ were obtained with the PBE functional, def2-SVP basis set and def2-J auxiliary basis set.

The next numerical test concerns the up-translation parameter *Lincr*. This parameter represents the increase of angular momentum at each level of the bubble hierarchy. Again, quick and stable convergence is observed and a value of *Lincr* = 6 appears to be well-converged while the total computation time is rather insensitive to the choice of this value (Figure 9). The reason for this behavior is, that most of the far-field time is spent computing bottom-level interactions. However, the total far-field time is much smaller than the near-field time as will be discussed in more detail below. A careful inspection also reveals that only the first up-translation is important for the accuracy of the algorithm and smaller values in the range 2–4 are acceptable for the higher bubble levels.

**Figure 10:**
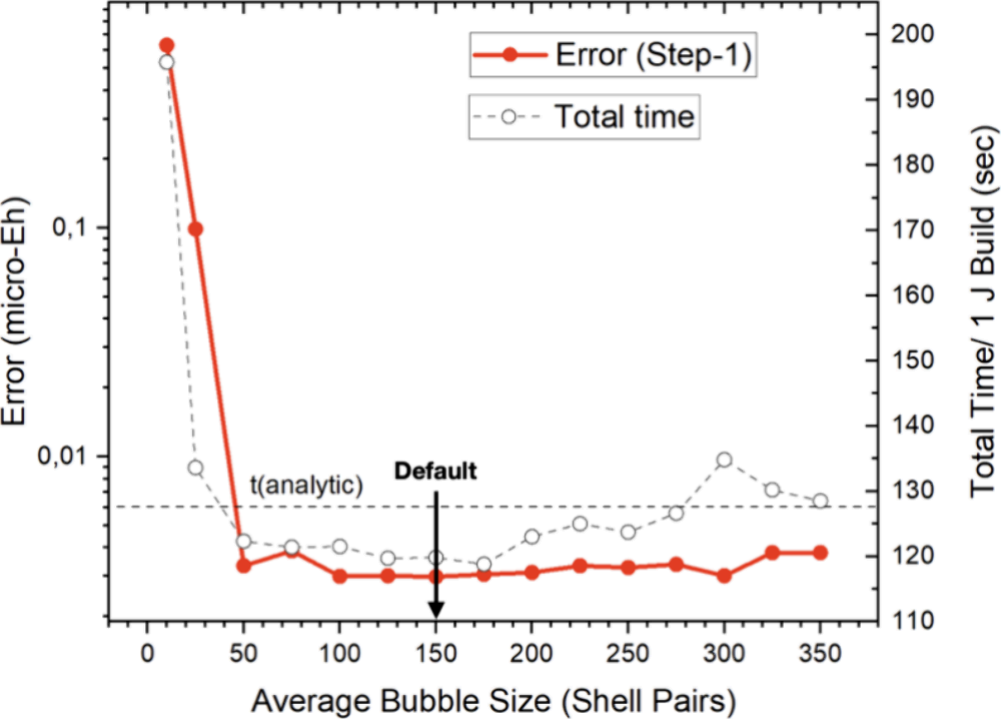
Convergence of the RI-BUPO-J method with respect to the target size of the shell pair bubbles. The red dots represent the error introduced by the multipole approximation, while the open circles represent the time required for one Coulomb build with the RI-BUPO-J algorithm. The horizontal dashed line is the time taken by the standard Split-RI-J algorithm. All results for (gly)_50_ were obtained with the PBE functional, def2-SVP basis set and def2-J auxiliary basis set.

The final test concerns the target size of the shell pair bubbles. The dependence of the results is shown in Figure 10. For very small bubbles, the far field is carved out of the molecular topology very accurately since the shell pair bubbles remain very small (about the size of the average shell pair extent). However, for very small bubbles, an insufficient number of interactions can be presummed and the large number of bubbles (thousands) starts to introduce overhead. On the other hand, if the bubble sizes become too large, the bubble radii will increase and the NF/FF separation will suffer. The results in Figure 10 clearly demonstrate that the dependence of the accuracy or timing on the bubble size is quite limited. We choose an average of 150 shell pairs per bubble as our default value since it appears to represent a reasonable compromise between the conflicting requirements discussed above.

### 4.2 Accuracy in Step 2

The final Coulomb assembly step was investigated in the same way as step 1 by performing step 1 analytically and only introducing the multipoles in the second step. We note in passing that the error introduced in steps 1 and 2 are very nearly additive such that there is no error “explosion” once the multipoles are introduced in both steps.

As mentioned above, for the second step, there are several options. The auxiliary shell bubbles can be created as “atomic bubbles” in which case the bottom-level multipoles are exact or as “molecular bubbles” which allow a larger number of auxiliary shells to be grouped together at the bottom level. Second, the expansion of the shell pair multipoles can be done at the contracted shell pair center (which is not exact but compact) or for each primitive pair individually (which is exact but not compact). It turns out that the most successful combination consists of the use of atomic auxiliary bubbles together with contracted shell pairs. This choice still leaves the expansion at the bottom level exact and short on one side while remaining compact.

**Figure 11:**
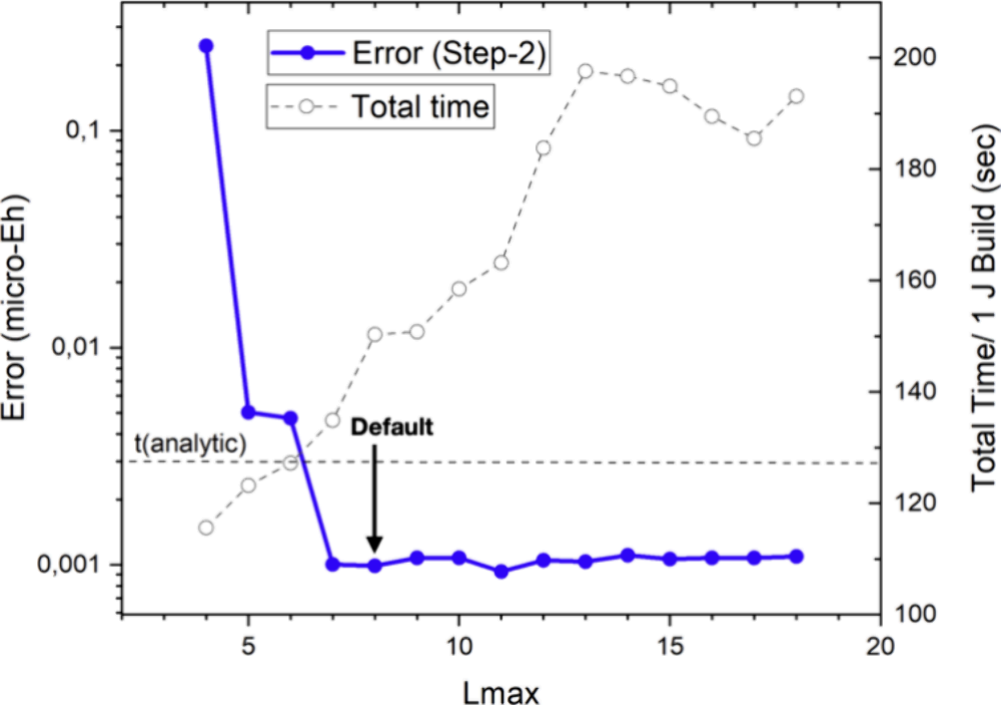
Convergence of the RI-BUPO-J method with respect to the expansion length of the contracted shell pair in the bra. The blue dots represent the error introduced by the multipole approximation, while the open circles represent the time required for one Coulomb build with the RI-BUPO-J algorithm. The horizontal dashed line is the time taken by the standard Split-RI-J algorithm. All results for (gly)_50_ were obtained with the PBE functional, def2-SVP basis set and def2-J auxiliary basis set.

In Figure 11, we show the convergence of the results with respect to the expansion length of the contracted shell pair. Again, full accuracy is readily reached and we choose a default value of Lmax = 6 for this step. Unlike step 1, it also becomes evident that the computer time is far more sensitive to this choice and that savings over Split-RI-J are more difficult to realize. This situation is undoubtedly due to the fact that the auxiliary-basis density that is multipole expanded in Step 2 is a far smaller object than the shell pair-based density matrix that is multipole expanded in Step 1. Thus, there is far less opportunity for multipole approximations to become effective for the final Coulomb assembly since, to some extent at least, the auxiliary-basis density in itself represents a form of atom-centered multipole expansion of the Coulomb interaction.

**Figure 12:**
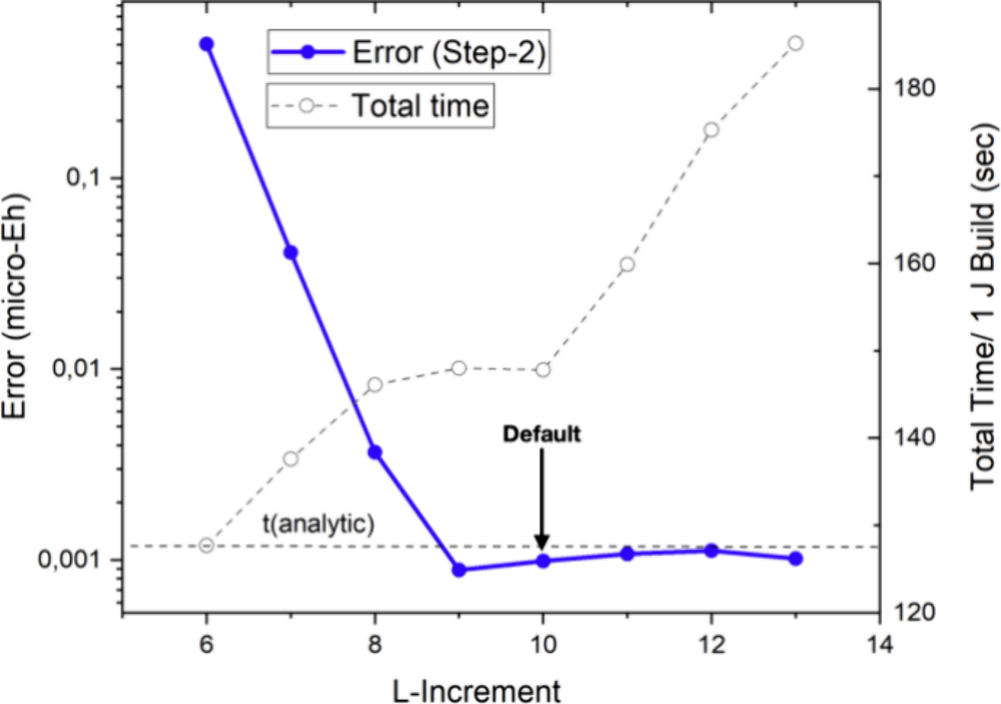
Convergence of the RI-BUPO-J method with respect to the up-translation increment from bottom-level atomic bubbles. The blue dots represent the error introduced by the multipole approximation, while the open circles represent the time required for one Coulomb build with the RI-BUPO-J algorithm. The horizontal dashed line is the time taken by the standard Split-RI-J algorithm. All results for (gly)_50_ were obtained with the PBE functional, def2-SVP basis set and def2-J auxiliary basis set.

A necessary consequence of using exact atomic bubbles on the bottom level of the expansion is that a large up-translation is required to guarantee the accuracy of the algorithm in Step 2. The results in Figure 12 demonstrate that a value of *Lincr* = 10 is necessary to reach full accuracy. Again, this large up-translation is only necessary of the first bubble layer and subsequent translations of the order of 2–6 are perfectly acceptable in order to maintain full accuracy.

In summary, it was demonstrated that using the RI-BUPO-J algorithm full numerical accuracy in the Coulomb interaction can be maintained while the initial results already indicate that computational savings over the highly efficient analytical Split-RI-J algorithm are possible.

### 4.3 Finding the Best Algorithm

In order to find the best compromise between efficiency and accuracy, we have defined a range of standard settings. Table 1 documents the settings and acronyms. We emphasize that we do not intend to define a dozen or more methods that only differ slightly in some technical settings but the intention of this section is to identify the best algorithm. After this has been accomplished, the many acronyms will be discarded and the most successful algorithm will be given the acronym RI-BUPO-J.

**Table 1:** Acronyms Used for Temporarily Defined Versions of the RI-BUPO-J Algorithm^a^

algorithm	Lmax	Lincr1	Lincr2	MP in RI/J 1st step	MP in RI/J 2nd step	incremental restart
RI-BUPO1/J	10	6	6	yes	yes	no change
RI-BUPO2/J	6	4	3	yes	yes	no change
RI-BUPO3/J	10	6	6	yes	no	no change
RI-BUPO4/J	6	4	3	yes	no	no change
RI-BUPO-5/J	10	6	6	yes	atomic	no change
RI-BUPO-6/J	6	4	3	yes	atomic	no change
RI-BUPO1S/J	10	6	6	yes	yes	exact build
RI-BUPO2S/J	6	4	3	yes	yes	exact build
RI-BUPO3S/J	10	6	6	yes	no	exact build
RI-BUPO4S/J	6	4	3	yes	no	exact build
RI-BUPO2A/J	6	4	3	yes	yes	accurate build
RI-BUPO4A/J	6	4	3	yes	no	accurate build

a Common settings: TScreen= 1e–10, Max no of multipole levels = 20, TargetDim1 = 150, TargetDim2 = 3, LAbsMax = 44. “Accurate build” means that upon incremental restart, the parameters of BUPO1 are chosen.

Below, we differentiate two different scenarios for the formation of a Coulomb matrix. In the “full build” for iteration *n*, a Coulomb matrix is formed by
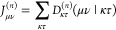
35

Or the RI-J equivalent of it, where ***D***^(*n*)^ is the density matrix at iteration *n*. Based, on Almlöf’s suggestion,^58^ it is customary to replace the density matrix by the difference density matrix between two iterations leading to the “incremental build.”
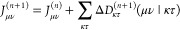
36With Δ***D***^(*n*+1)^ = ***D***^(*n*+1)^ – ***D***^(*n*)^. The objective of this construction is that Δ***D*** approaches the **0**-matrix as the calculation approaches convergence and consequently, prescreening based on the difference density matrix becomes more effective than prescreening based on the full density matrix. Since this procedure also leads to the accumulation of numerical noise, it is advisable to restart the procedure with a full Fock matrix build every, say, ten to 20 cycles. Below we refer to “Incremental Restart” when the Fock matrix is rebuilt with a full density matrix. This happens automatically at the first SCF iteration and then whenever the number of iterations hits a multiple of the restart cycle (we choose ten iterations in the calculations reported in the remainder of the paper). Thus, in a calculation that converges after, say, 21 cycles, there would be three full Coulomb/Fock matrix builds involved.

Specifically, we have investigated the following variations of the algorithm:

- Two different combinations of multipole settings for “high” and “low” accuracy are defined in Table 1. The term “Accurate build” refers to a BUPO J-matrix build with the high-accuracy settings. “No change” means that the incremental restart J-matrix build do not change any multipole settings and hence, every multipole J matrix is built with the same truncation parameters.
- Possible replacement of the BUPO Coulomb matrix assembly with the analytic procedure from the Split-RI-J approach upon incremental restart. This is referred to as “Exact build” and leads to quadratic scaling.
- Possible “multipole acceleration” by performing the Coulomb matrix assembly in terms of atomic multipole moments. In this case, there is no hierarchical treatment of multipoles and the far-field part of the calculation simply uses the multipole approximated integrals in favor of the analytic ones. Multipoles are calculated for each atom separately. This is similar to the “multipole-accelerated RI-J“ (MARIJ) method of Ahlrichs and co-workers^24^ and leads to quadratic scaling. This is referred to as “Atomic” in Table 1.
- Not using the multipole approximation in either the first or second step of the RI-J procedure and use the Split-RI-J instead. These are the columns “MP in RI/J1^st^/2^nd^ step.”

The results of these calculations show that all RI-BUPO-J versions easily reach and surpass micro-Eh accuracy in the total energies which is considered to be satisfactory (Table 2). The two most efficient variants are RI-BUPO2/J and RI-BUPO2A/J. These methods are based on the “lower accuracy” multipole settings with an initial Lmax of 6. The algorithms only differ in the details that upon incremental restart, RI-BUPO2A/J switches to the “high accuracy” multipole settings with Lmax = 10 while RI-BUPO2/J performs all SCF cycles with the same settings. That the full Fock build with the more accurate multipole settings is definitely worthwhile is seen by the drastic error reduction by a factor of 10 while the total time increase is only a few seconds. Furthermore, further investigations have shown that without the accurate rebuild RI-BUPO2/J can become numerically unstable for very large systems. None of the variants that break linear-scaling, either by doing a full analytic J-matrix build in the final step of the RI procedure (RI-BUPO*S/J), nor the variants that use “multipole acceleration” using atomic bubbles (RI-BUPO5,6/J) offer any advantages and lead to less efficient calculations with no improvements in the accuracy. We conclude that RI-BUPO2A/J is the preferred algorithm and we will henceforth refer to this algorithm simply as “RI-BUPO-J.”

**Table 2:** Comparison of Different RI-BUPO-J Algorithms Relative to Traditional RI-J and Split-RI-J^a^

algorithm	error (μEh)	time T/s	time J/s
RI-J		976.6	783.1
Split-RI-J		427.3	230.5
RI-BUPO1/J	0.00	610.3	416.9
**RI-BUPO2/J**	**0.10**	**488.2**	**294.5**
RI-BUPO3/J	0.01	545.2	351.3
RI-BUPO4/J	0.01	517.8	324.2
RI-BUPO5/J	0.01	660.4	468.4
RI-BUPO6/J	0.02	577.5	384.5
RI-BUPO1S/J	0.01	626.3	432.6
RI-BUPO2S/J	0.01	520.6	326.5
RI-BUPO3S/J	0.01	571.5	377.2
RI-BUPO4S/J	0.01	545.3	352.0
**RI-BUPO2A/J**	**0.01**	**498.5**	**304.3**
RI-BUPO4A/J	0.01	520.7	327.7

a Time T refers to the sum of all times for the entire solution of the SCF equations excluding the initial guess and grid setup. Time J is the accumulated time for all 17 Coulomb matrix builds that were required to converge the PBE/def2-SVP DFT calculations. 16 cores were used throughout. All calculations converged to 10^–9^ Eh. The system was gly_50_ with 353 atoms, 3574 basis functions, 11,521 auxiliary functions.

### 4.4 Relative Energies

**Figure 13:**
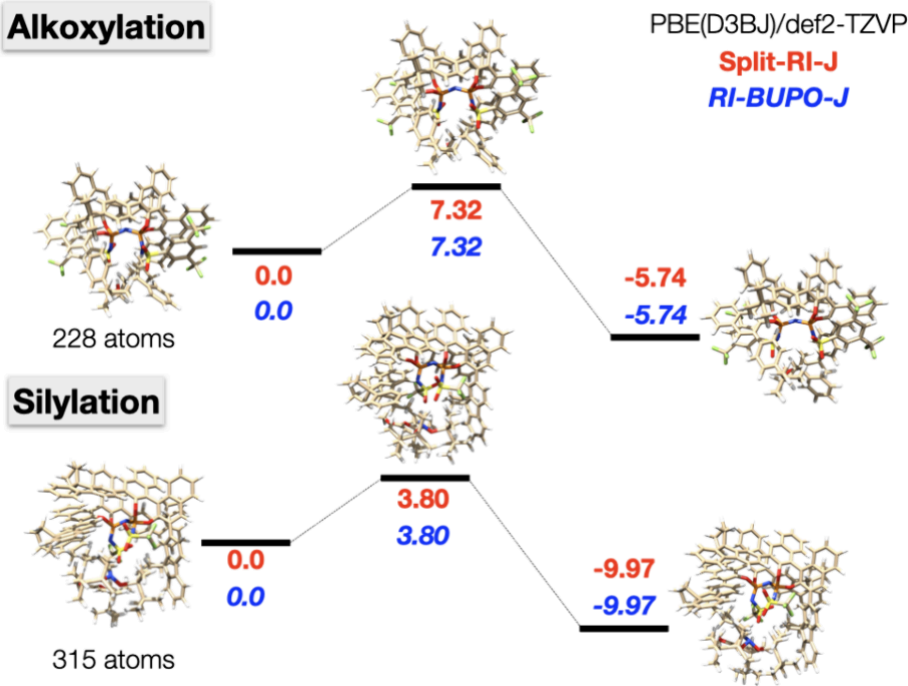
Evaluation of relative energies using the RI-BUPO-J algorithm relative to the same calculation using Split-RI-J. Calculations were done with the def2-TZVP basis set, the def2/J auxiliary basis set, and the PBE functional with no dispersion correction. The ORCA VeryTightSCF settings were applied which converges the self-consistent field equations to about nEh accuracy. For both reactions, reactants (left), transition state (middle) and product (right) are shown. The structures for reactions were taken from ref (62, 63).

Given the fact that the absolute accuracy of the BUPO algorithm compared to RI-J or Split-RI-J with the same basis set and auxiliary basis set is on the order of nEh to μEh, it is not to be expected that relative energies show chemically significant errors that could be attributed to the multipole approximation. We have nevertheless investigated two organocatalytic reactions studied in recent work.^62,63^ The results shown in Figure 13 demonstrate that the energies obtained with the RI-BUPO-J approximation are indistinguishable from the results obtained with Split-RI-J. We note in passing that these systems are too small for the RI-BUPO-J approximation to lead to timing advantages. The calculations with Split-RI-J are about 25% faster.

### 4.5 Scaling

In order to measure the computational scaling of the RI-BUPO-J algorithm with respect to system size, we have employed the same glycine chains that were used for the accuracy tests. Measuring accurate, fair and reproducible timings has turned out to be challenging given the complex acceleration and scheduling algorithms that modern operating systems perform in order to maximize overall machine performance. All calculations reported in the remainder of the paper (except the parallel scaling calculations below) used the same machine and were performed on 16 cores and a maximum of 16 GB of main memory per core unlike otherwise noted. The machine is a Threadripper PRO 7955WX 16-Cores; 1 socket machine with 512GB RAM, 8TB NVMe SSD scratch. BLAS operations used OpenBLAS 0.3.29, parallelization via openmpi 4.1.7 using scalapack 2.2.2.

**Figure 14:**
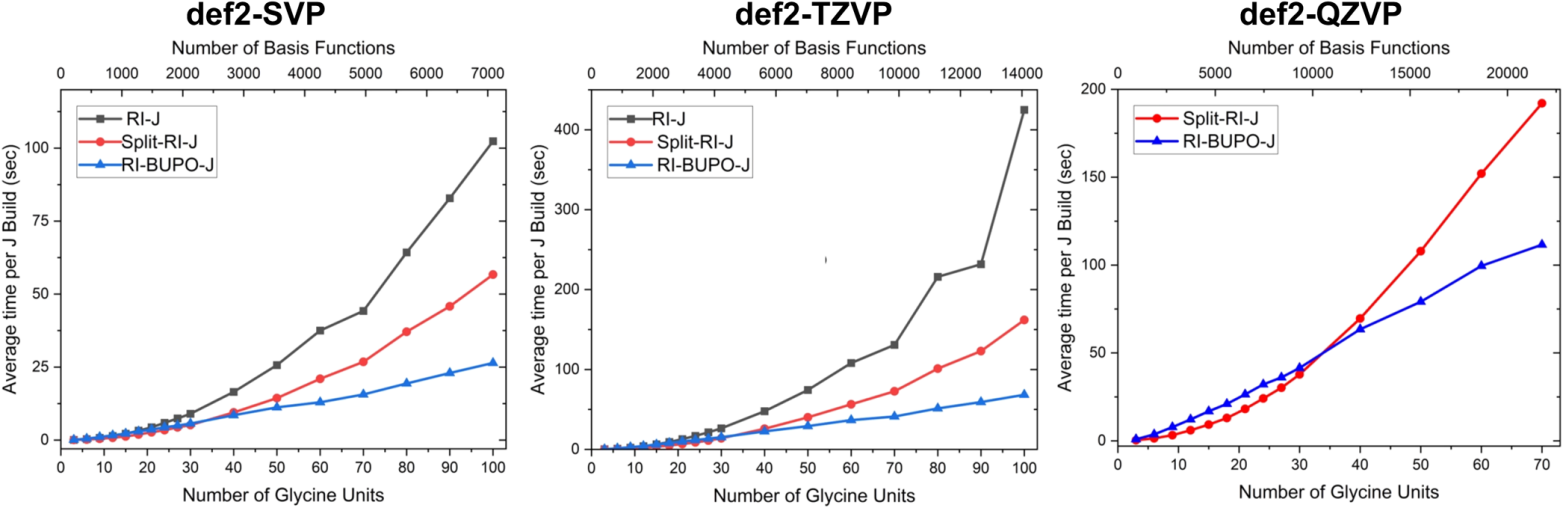
Scaling of the RI-BUPO-J algorithm compared to Split-RI-J (Single core of an Apple M3 processor). Times are in seconds and normalized to the number of SCF cycles.

In Figure 14 we compare the scaling obtained with the RI-BUPO-J, Split-RI-J and traditional RI-J algorithms. We also compare the def2-SVP, def2-TZVP and def2-QZVP basis sets respectively. In order to reach a meaningful comparison, the times were normalized by the number of SCF iterations.

All calculations were converged to an accuracy of 1e-9 Eh. All BUPO energies match the Split-RI-J energies to an accuracy of 1e-6–1e-9 Eh.

Based on the results shown in Figure 14, both Split-RI-J and RI-BUPO-J are always faster than the traditional RI-J calculation by a factor of 2–3. RI-BUPO-J shows linear-scaling with system size with a crossover relative to Split-RI-J at about 40 glycine units which has 283 atoms. With larger basis sets, the crossover appears to occur slightly earlier but remains in the same ballpark. Please note that a single Coulomb matrix build for (gly)_100_ with more than 7000 basis functions in the def2-SVP case takes about 25 s and in the case of def2-TZVP with more than 14000 basis functions that time is only about 60 s. In the largest system treated with def2-QZVP, (gly)_70_ with about 22000 basis functions, the average time per Coulomb build is still less than 120 s (two minutes). In fact, we show below that this performance is so high that other parts of the algorithm become rate limiting.

**Figure 15:**
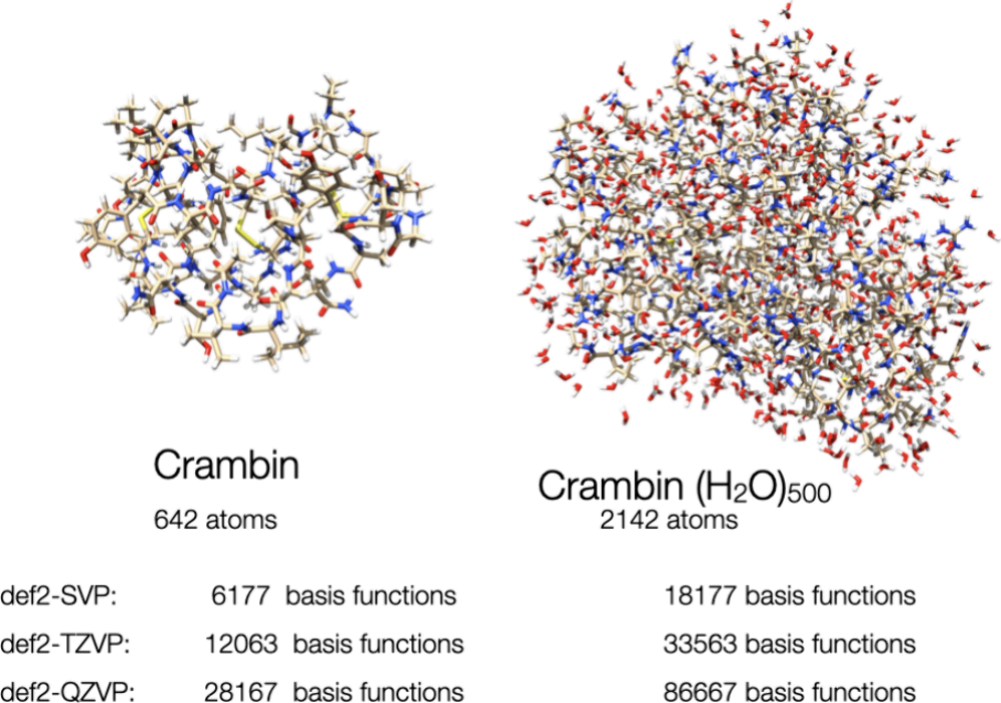
Structures of the crambin molecule and crambin (642 atoms) solvated with 500 water molecules (2142 atoms) together with the number of basis functions for all-electron DFT calculations.

For real, three-dimensional systems, we expect the crossover to happen at larger system sizes. It is, however, encouraging that the overhead created by the BUPO algorithm for smaller system sizes appears to be small such that for smaller systems, RI-BUPO-J and Split-RI-J can be used essential interchangeably without a loss in accuracy or an undue decrease of performance.

### 4.6 Large Three-Dimensional Systems

As an example for more spherical, three-dimensional systems, we have treated the crambin molecule (642 atoms) as well as a version of crambin explicitly solvated with 500 water molecules (2142 atoms), both optimized with the GFN-FF force field. All coordinates are found in the SI.

In order to show that such large calculations are possible with our code, we have treated both crambin and crambin(H_2_O)_500_ with three different basis sets of increasing size, namely def2-SVP, def2-TZVP and def2-QZVP. These calculations feature over 2000 atoms and over 30,000 basis functions (Table 3). The very largest calculation–crambin(H2O)_500_/def2-QZVP–failed with the present code due to memory constraint in the startup phase of the calculation, but we anticipate that it will also become possible with a few technical improvements in the infrastructure. We observe that a roughly spherical molecule, like crambin with 642 atoms, is still too small for RI-BUPO-J to win over Split-RI-J. However, for crambin (H_2_O)_500_, the situation is at least partially reverse and the time required for RI-BUPO-J is lower than that for Split-RI-J with the small def2-SVP basis set. We should note, however, that for these very large calculation with around 30000 basis functions, the Fock (Kohn–Sham) matrix formation is not the rate-limiting step in the calculation and only accounts for about 20% of the SCF time. Rather 70% or more go into the solution of the SCF equations using a DIIS or approximate second-order (SOSCF) algorithm, and 5% go into the calculation of the density matrix, all of which are cubically scaling. Hence, before optimizing any Fock matrix formation algorithm any further, it will be necessary to improve on the efficiency of the solvers. It is nevertheless gratifying to see that the RI-BUPO-J approximation remains accurate to better than 1 mEh even for the largest calculations. The calculation on crambin(H_2_O)_500_ with the def2-QZVP basis could not be done on our machine with 512 GB main memory due to technical limitations that are unrelated to the Fock matrix construction algorithm.

**Table 3:** Results of All Electron Calculations on the Crambin Protein and Crambin(H_2_O)_500_ Using the PBE Functional, the DEF2-MTZVP/J Fitting Basis Set and the Indicated Basis Set^a^

	**basis set**	**numberof cores**	**Split-RI-J (s)**	**RI-BUPO-J (s)**	**error (mEh)**
crambin	Def2-SVP	16	37.7	46.9	0.001
	Def2-TZVP	16	43.4	124.8	0.000
	Def2-QZVP	16	127.7	366.8	0.079
crambin (H_2_O)_500_	Def2-SVP	16	379.1	298.1	0.024
	Def2-TZVP	12	399.8	1019.3	0.704

a The number of basis functions and atoms are shown in Figure 15. The times for Split-RI-J and RI-BUPO-J refer to the average time for one Coulomb matrix build. (The error is the absolute error with respect to Split-RI-J).

### 4.7 Parallelization Efficiency

**Figure 16:**
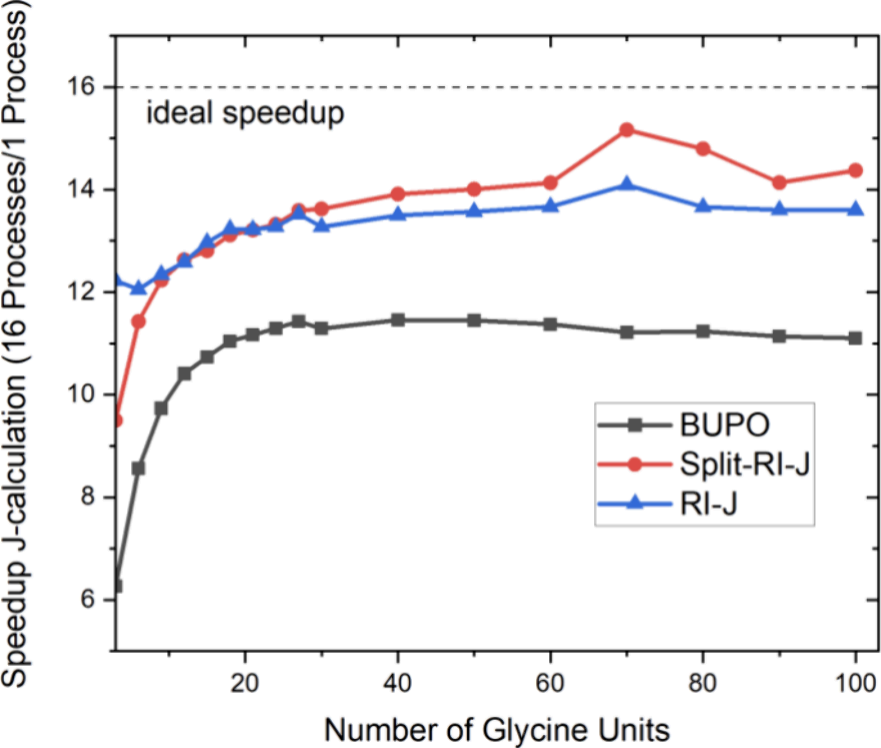
Parallelization efficiency of the RI-BUPO-J algorithm relative to traditional RI-J and Split-RI-J. What is plotted is the ratio of the same PBE/def2-SVP calculation on (gly)_n_ with 16 processes versus a single process. The ideal speedup would therefore be 16.

In Figure 16, we show the speedup for the J-matrix construction of the RI-BUPO-J method relative to Split-RI-J and traditional RI-J using 16 cores of an older test machine versus one core on the same machine (AMD EPYC 7302P 16-Core Processor with 256GB RAM and a 1.5 TB HDD scratch disk).

It is evident, that both Split-RI-J and traditional RI-J parallelize with slightly better efficiency reaching average speedups of 13–14 whereas RI-BUPO-J reaches a steady speedup of around 11. We attribute this to the higher algorithmic complexity of the BUPO algorithm that introduces slightly more overhead and need for more frequent synchronization relative to more traditional RI-J algorithms. Thus, improvement of the parallel scaling with respect to the number of processes is an obvious target for further improvement.

### 4.8 Detailed Timing Analysis

In order to obtain some insight into the relative importance of the individual steps involved in the RI-BUPO-J algorithm, we provide a breakdown of the relevant computational steps for the first and second iteration of the PBE/def2-SVP calculation on gly_100_ on a single Apple M3 processor. The first and second iteration differ in that in the first iteration a full J-matrix build is performed using the “accurate” multipole settings (Table 2) while in the second iteration an incremental build with the “reduced accuracy” multipole settings is performed.

The calculation begins with a step that is only performed once in the entire calculation and that consists of creating the bubble hierarchies for both, shell pairs and auxiliary functions. This step only takes 3.5 s and is therefore negligible. The algorithm finds eight shell pair and five auxiliary shell bubble levels for this system. The sizes, average number of objects are documented in Table 4.

**Table 4:** Bubble Hierarchy for Gly_100_/def2-SVP Given for Shell Pairs/Auxiliary Shells^a^

level	no. bubbles	radius (Bohr)	av. dimension
1	2825/703	11.91/12.77	150.0/10.7
2	941/117	14.0/14.4	3.0/6.0
3	313/19	17.7/23.9	3.0/6.0
4	104/3	24.2/69.1	3.0/6.0
5	23/1	33.1/196.8	3.0/6.0
6	11/–	45.8/–	3.0/–
7	3/–	90.0/–	3.0/–
8	1/–	202.0/–	3.0/–

a The average dimensions are input parameters that the bubble construction algorithm can take some liberty with but does respect nearly perfectly. The radius is determined by the shell pair or auxiliary shell center plus the extent of the shell pair or auxiliary shell. Note that the average dimension on the bottom level (level 1) is the average number of shell pairs/auxiliary shells in each bubble while subsequent levels refer to the number of sub-bubbles in each bubble of the next hierarchy.

The timing breakdown of the RI-BUPO-J algorithm in comparison to Split-RI-J (Table 5) reveals some interesting results. In the first step (the projection of the density onto the auxiliary basis, eq 9), the calculation is dominated by the near-field time and the calculation of the multipole moments takes a non-negligible time. In the final step (J matrix assembly), the situation is reversed and the far-field time still dominates over the near-field time, despite the large system size while the calculation of the multipole moments over auxiliary functions is computationally negligible. The reason for this behavior is simply that in the first step one expands shells pairs in multipoles and in the second step one expands auxiliary shells in multipoles. Since there are far more non-negligible shell pairs (423,750) than auxiliary shells (7522) the benefits of the multipole approximation are necessarily higher in the first step than in the second. In addition, the second step requires the multipole expansion of each shell pair to be calculated individually for the far-field part which is distinctly more expensive than the calculation of the multipole expansion for an entire bubble encompassing an average of 150 shell pairs in step 1.

**Table 5:** Breakdown of the Timings for the RI-BUPO-J Algorithm for the First Two SCF Iterations on Gly_100_/def2-SVP (703 Atoms, 7124 Basis Functions, 22,971 Auxiliary Basis Functions)^a^^,^^b^

	iteration 1		iteration 2	
step	RI-BUPO-J	split-RI-J	RI-BUPO-J	split-RI-J
total time step-1	**136.2**	**215.4**	**123.9**	**205.5**
time (near field)-1	115.9	215.4	108.8	205.5
time (far field)-1	10.1	0.0	10.2	0.0
multipole calculation	8.2	0.0	2.9	0.0
number near-field batches	9.0%	74.2%	8.6	71.4%
number far-field batches	88.1%	0.0	88.1	0.0
number skipped batches	2.9%	25.8%	3.3	28.6%
time (linear equations)	**0.9**	**0.9**	**0.9**	**0.9**
total time step-2	**99.9**	**154.4**	**66.9**	**144.3**
time (near field)-1	32.8	154.4	32.1	144.3
time (far field)-1	65.1	0.0	32.9	0.0
multipole calculation	0.07	0.0	0.04	0.0
number near-field batches	10.2%	90.9%	9.6%	87.0%
number far-field batches	88.9%	0%	89.7%	0%
number skipped batches	0.9%	9.1%	0.7%	13.0%
total time for J matrix	**237.1**	**355.5**	**191.9**	**350.7**

a Given this analysis of the timing breakdowns it is apparent, that there is further room for improvement.

b Step-1,2 refer to eqs 9 and 11, respectively. Timings are in second and number of batches is given in per-cent of the total number of batches processed.

We emphasize that the solution of the linear equations via the Cholesky decomposition of the V-matrix, despite its higher scaling, only takes 0.9 s in this example. This demonstrates that any deviation of the algorithm from linear-scaling is definitely not coming from this step since it remains negligible for at least 1000 atoms, probably even up to much larger sizes. We note in passing that multipole translation is a computationally completely negligible step in the RI-BUPO-J algorithm. For example, it accounts for just 0.9 s out of 252 s for the first iteration on gly_100_. This appears to be a rather distinctive difference to other multipole algorithms that are sometimes reported to be dominated by the time required for multipole translation.^64^

The comparison between Split-RI-J and BUPO-J is also interesting. The saving due to the multipole approximation in the first step are on the order of 35–40% and do not depend much on the multipole accuracy settings. This makes sense, since the calculation time is dominated by the near-field part of the calculation that is reduced from 74.2% analytic (near-field) batches in Split-RI-J to only 9.1% analytic (near-field) batches in RI-BUPO-J. One can also see how the multipole approximation replaces prescreening with summation by considering that Split-RI-J screens 25.8% of all batches while RI-BUPO-J only screens 3%.

The savings due to the more aggressively truncated multipole approximation are much more effective in the second step where the more accurate settings in iteration one lead to savings of only about 30% while for the second iteration these savings double. Hence, in principle, the reduced accuracy settings are only effective for the second part of the RI-BUPO-J calculation and according to the results in Section 4.2, this is also the most critical step for the overall accuracy of the approximation. As indicated by the results in Section 4.3, it is still important to perform this step with a hierarchical multipole expansion since replacing it by either the fully analytic calculation or an atomic multipole-based “multipole accelerated” procedure is distinctly less efficient.

It is striking that in Step 1 the number of near-field batches is more than 1 order of magnitude smaller than in the Split-RI-J case but the time to compute these far fewer batches is only a factor of 2 smaller than the calculation of Step 1 in the Split-RI-J case that treats all shell pairs analytically. This happens despite the fact that the very same integral routines are called in both cases. We have searched intensely for the origin of this observation. It is most likely related to overhead related to fetching the shell-pair data required for the calculation of the analytic integrals out of very heterogeneous memory locations. Consequently, we have investigated algorithms in which the shell pair data are recomputed on the fly and variants in which the shell pair data of the surviving near-field shell pairs are preorganized in a contiguous memory location after the far-field calculation. Neither attempt was successful in fixing the problem. Thus, unfortunately, we currently have no satisfactory explanation for this phenomenon but we obviously hope to significantly speed up the algorithm once the source of this slow-down (on a per-integral batch) in the near-field part of the expensive first step is found.

In the second step of the procedure, there probably is not too much more room for improvement since the multipole truncation is already handled as aggressively as possible without compromising the numerical stability of the algorithm for big systems.

We have carefully optimized the algorithms for the calculations of the multipole integrals and the interaction tensor with respect to FLOP count and memory access. The actual multipole interaction is then performed purely in real algebra by BLAS operations which drive the computer at peak speeds.

## 5 Discussion

In this paper, we have developed the “Bubblepole” (BUPO) algorithm, an asymptotically linear-scaling, hierarchical multipole-based algorithm that can be used in many ways but has been used here for the formation of Coulomb matrices in self-consistent field calculations. The algorithm can be applied with or without the RI/DF approximation. We have only documented the RI-version of the algorithm here since it holds the greatest potential for practical applications. The conclusions will, however, largely carry over to the non-RI version as well.

The BUPO-J algorithm shares with FMM approaches the hierarchical treatment of multipoles that are being translated from one recursion level to the next. However, it differs strongly from the FMM by not using a boxing algorithm but instead is based on the grouping of spatially close objects such as shell pairs, auxiliary shells or point-charges in spheres (bubbles) that fully enclose them. This allows the algorithm to concisely adapt itself to the molecular topology while keeping an even workload across all layers of the hierarchy. In addition, we have introduced a straightforward algorithm to compute shell pair extents that leads to a very accurate division between near-field and far-field contributions. In our opinion, this definition of shell pair extents has advantages over earlier proposals because the extents for distant, weakly overlapping two-center shell pairs gets *smaller* with increasing intercenter distance while in other proposals the extents will get *larger*, which we regard as unphysical.

The construction of the multipole hierarchy in terms of bubbles is fairly different from traditional boxing algorithms. In boxing algorithms, the space is evenly divided. This means that the molecule has a rather arbitrary orientation in the box. Depending on the arbitrary rotational degrees of freedom, one might obtain slightly different results which means that boxing algorithms are not strictly rotationally invariant. It may well be that actual implementations of the existing FMM algorithms have taken appropriate measures to make them rotationally invariant but such details are often not reported in publications. In boxing algorithms, the amount of content in each box remains largely uncontrolled and box boundaries may arbitrarily cut through bonds or atoms. Leakage of density outside the box borders must be dealt with. On the other hand, the bubbles are constructed in a way that they are shape adapting and of nearly constant size. This guarantees a very balanced and even treatment that lends itself well to parallelization. In addition, bubble boundaries are strictly enclosing their objects with no leakage possible. Thus, the bubble hierarchy may be considered as a variant of the FMM hierarchy that is, in our opinion, better adapted to chemistry and the shape of molecules. This may well be different for the case of electrostatic embedding of a molecule in a point-charge field, as discussed recently.^65^

We have numerically demonstrated that the RI-BUPO-J algorithm is linear-scaling with respect to system size. The algorithm is highly efficient and, at the same time, also numerically accurate. As documented above, μEh to nEh accuracy (relative to Split-RI-J) has been achieved even for the largest systems studied here with about 1000 atoms. While the algorithm will outperform most other Coulomb algorithms, the competition with the Split-RI-J algorithm is stiff given that the latter is an already highly efficient and optimized way to build Coulomb matrices. It remains the most efficient way to calculate Coulomb matrices for most three-dimensional molecules and linear molecules with, perhaps, up to 300 or 400 atoms. The savings offered by the linear-scaling treatment only start to be effective and lead to tangible savings beyond this size. However, as witnessed by the example of crambin(H_2_O)_500_, if one reaches molecular sizes of thousands of atoms and tens of thousands of basis functions, the Fock matrix construction may no longer be the most expensive step and other steps that are more steeply scaling become dominant, for example the calculation of the density matrix or the orbital update. Finding efficient and robust algorithms for these steps will therefore be a future research focus that will likely lead to tangible acceleration of very large SCF calculations.

It should be realized, that approximating the Coulomb energy (and the Coulomb contributions to the Fock or Kohn–Sham matrix) in SCF calculations can probably be regarded as a worst-case scenario for a multipole-based algorithm. For large molecules, the Coulomb energies are on the order of hundreds or thousands of Hartrees while the required accuracy is in the range of micro-Eh. Thus, the algorithm is forced to produce approximations with at least 11 or 12 significant figures which is no small burden. In addition, the Coulomb interaction in the self-consistent field is the most direct form of an electrostatic interaction possible. Here, the density self-interaction decays only as 1/*R* which means that the interactions are large and of long-range character. We believe that in practically any other application of the multipole approximation, it will be far easier to reach sufficient accuracy while offering much larger potential for significant speedups. Thus, extensions to molecular gradients, response properties, excited states, exact exchange, embedding potentials or implicit solvation are self-evident and will be reported in due course. In addition, it is, of course, well-known that the multipole approximation comes to full fruition when combined with electron correlation treatments where the leading long-range term is the dispersion interaction that decays as R^–6^. The BUPO approximation lends itself particularly well to this application as will be explored in detail in future work.

## Appendix: Algorithm for multipole integrals

In this appendix, we are studying the following integral (***C*** is the desired expansion point for the multipoles):

37

*L* = *e* + *f* + *g* is the requested order of the multipole expansion and *G*_μ_(***r****_A_*) (centered at point ***R****_A_*) and *G*_ν_(***r****_B_*) (centered at point **B**) are the two Gaussians involved in the shell pair (***r****_X_****= r–R****_X_****;****X****=****A*, *B*). It is obviously advantageous to calculate all integrals with *L* = 0 to *L* = *L*_max_ = *e* + *f* + *g* at the same time in order to avoid redundant computation. The equations below should not be considered as new but rather serve as a documentation of our implementation. We refer to refs (52, 53) for more details and derivations.

The Gaussian product is expanded as usual:

38

With *t* + *u* + *v* ≤ *l*_μ_ + *l*_ν_. *E*_*tuv*_^μν^ are the Hermite expansion coefficients in the McMurchie-Davidson method^40^ and Λ_*tuv*_(***r****_P_*) is a product of three on-dimensional Hermite Gaussians (vide infra) centered at the Gaussian product point ***P*** (28).

Inserting 38 into 37 gives

39

The Hermite Gaussians are defined as

40

Obviously, the Hermite Gaussian factorize like Λ_*tuv*_(***r****_P_*) ***=*** Λ_*t*_(*x*_*P*_)Λ_*u*_(*y*_*P*_)Λ_*v*_(*z*_*P*_). This means that the multipole integrals factorizes into Cartesian directions and we can focus on a one-dimensional integral:

41

The quantities *M*_*t*_^*e*^(***C***) can be viewed as one-dimensional Hermite multipole integrals:

42

They can be calculated in a recursive manner. The starting point for the recurrence is simply:

43

By means of the properties of the Hermite polynomials we furthermore have

44

Now one can write

45

Hence: *x*_*C*_***= P****_*x*_****–C****_*x*_* + *x*_*P*_ and we also write *X*_*PC*_***= P****_*x*_****–C****_*x*_*. That means that the integral *M*_0_^1^: can be expressed as

46

More generally, the recurrence relation for the Hermite Gaussian multiplied by *x*_P_ is

47

Thus, giving the recursion relation:

48

In general, integrals with *t* > *e* will vanish but the term with *t* = *e* needs to be calculated.^52,53^ Hence, we need to generate all terms up to *t* = *e*, even if the basis function has angular momenta lower than e.

The desired Cartesian multipole integral 37 is eventually calculated as

49

Or, more explicitly:

50

where the sums run from 0 to the smaller of the sum of the Cartesian components (e.g., *t*^μ^ + *t*^ν^) and the multipole order (e.g., *e*). Contracted integrals are obtained by simple summation over primitive pairs. This algorithm produces Cartesian multipole integrals over contracted spherical harmonic Gaussian shell pairs. These integrals are then transformed into the spherical harmonic basis in order to produce the integrals 19 that are used in the BUPO algorithm.

## Supporting Information Available

Structures, alkoxylation, crambin, glycine-chains, and silylation (ZIP)
